# Establishment of a Model to Predict the Prognosis of Endometrial Carcinoma Using Tumor‐Infiltrating Lymphocytes Evaluated With Artificial Intelligence: A Retrospective Analysis

**DOI:** 10.1002/cnr2.70535

**Published:** 2026-04-09

**Authors:** Taira Hada, Morikazu Miyamoto, Takahiro Einama, Soichiro Kakimoto, Makiko Koga, Takanori Watanabe, Yuka Otsuka, Jin Suminokura, Tsubasa Ito, Naohisa Kishimoto, Risa Tanabe, Soko Nishimura, Kento Kato, Hiroaki Soyama, Kohei Omatsu, Yoshinobu Hamada, Kimiya Sato, Masashi Takano

**Affiliations:** ^1^ Department of Obstetrics and Gynecology National Defense Medical College Hospital Tokorozawa Saitama Japan; ^2^ Department of Surgery National Defense Medical College Hospital Tokorozawa Saitama Japan; ^3^ Department of Obstetrics and Gynecology Self‐Defense Force Central Hospital Tokyo Japan; ^4^ Department of Basic Pathology National Defense Medical College Tokorozawa Saitama Japan; ^5^ Department of Clinical Oncology National Defense Medical College Hospital Tokorozawa Saitama Japan

**Keywords:** artificial intelligence, endometrial carcinoma, mismatch protein repair, prognosis, tumor‐infiltrating lymphocytes

## Abstract

**Background:**

The objective of this study was to establish a new model for predicting the prognosis of endometrial carcinoma (EC) using tumor‐infiltrating lymphocytes (TILs) based on artificial intelligence (AI).

**Methods:**

Patients with EC who were treated between 1989 and 2022 were included in this study. For each patient, one hematoxylin and eosin‐stained slide containing the most invasive frontline of the tumor was selected and digitized. The area within a 500 μm width span, extending 250 μm toward the stroma and tumor from the manually annotated invasive frontline, was automatically annotated. The average number of lymphocytes per area (μm^2^) in the annotated area was calculated using AI. Patients were classified into the High‐TIL and Low‐TIL groups, and survival analysis was conducted. Four mismatch repair (MMR)‐related proteins were evaluated using immunohistochemical staining.

**Results:**

A total of 659 patients were included: 346 (52.5%) in the High‐TIL group and 313 (47.5%) in the Low‐TIL group. MMR deficiency was observed more frequently in the High‐TIL group than in the Low‐TIL group (*p* < 0.01). Progression‐free survival (PFS) and overall survival (OS) were better in the High‐TIL group than in the Low‐TIL group (both *p* < 0.01). Multivariate analysis revealed that TIL status was a prognostic factor for PFS (hazard ratio [HR] (95% confidence interval [CI]) 0.61 (0.43–0.87); *p* < 0.01) and OS (HR (95% CI) 0.54 (0.33–0.86); *p* = 0.01).

**Conclusion:**

TILs evaluated using AI could accurately and significantly predict the prognosis of EC. Further studies are needed to establish new methods for evaluating TILs in ECs.

## 1 Introduction

Endometrial carcinoma (EC) is the most common gynecological carcinoma and accounts for 4.3% of carcinomas in women worldwide [1]. Total hysterectomy with bilateral salpingo‐oophorectomy is the primary surgical intervention for EC, and pelvic and para‐aortic lymphadenectomy can be selected based on the disease status [2]. Thereafter, adjuvant therapies such as radiation or chemotherapy are administered according to the degree to factors associated with recurrence, including age, lymphovascular invasion, tumor size, lower uterine segment or surface glandular involvement, myometrial invasion, and histological subtype [3, 4, 5, 6].

The presence of tumor‐infiltrating lymphocytes (TILs) has been identified as a prognostic factor for several types of carcinomas, including esophageal, breast, prostate, colon, and ovarian carcinoma [7, 8, 9, 10, 11, 12]. Furthermore, TILs were found to correlate with the clinical outcome of EC [7, 13, 14, 15, 16]. Conversely, TIL status was shown to be associated with mismatch repair (MMR) status [17]. According to the guidelines for TIL assessment based on the International Immuno‐Oncology Biomarker Working Group, the area of evaluation for TILs of ECs was defined as only within the tumor area, from the invasive frontline or within 1 mm from the tumor cell nest in the stromal area [7]. However, guidelines regarding the area for TIL evaluation or methods for lymphocyte enumeration are lacking, necessitating the development of standard models to evaluate TILs in EC.

Recent research has extensively focused on the effectiveness of artificial intelligence (AI) in pathological diagnosis [18, 19, 20, 21, 22]. AI can be applied in pathological diagnosis at various points, such as to enhance work efficiency, reduce discrepancies in diagnosis among observers, and quantify diagnosis and evaluation. For example, the efficacy of automated deep learning algorithms for detecting lymph node metastasis has been reported in the field of breast carcinoma [19]. In esophageal and colorectal carcinoma, the effectiveness of deep learning algorithms has been demonstrated for the detection of desmoplastic reactions [21, 22]. In the field of gynecological carcinoma, an AI‐based comprehensive evaluation of intraepithelial and stromal TILs of ovarian high‐grade serous carcinoma has been reported [23]. However, reports on the use of AI in the field of EC are lacking.

Therefore, in this study, we aimed to establish a new AI‐based model for TILs in the area around the invasive frontline of EC.

## 2 Methods

Patients with EC who underwent primary surgery between 1989 and 2022 at the National Defense Medical College Hospital were included in this study. Patients without clinical information or surgical tissue samples, as well as those with a prior history of chemotherapy, were excluded. Clinical information was collected from the medical records. We conducted pathological reviews using the 2020 World Health Organization criteria and classified these histological subtypes based on the 2023 International Federation of Gynecology and Obstetrics (FIGO) criteria [24, 25]. Non‐aggressive types were defined as grade 1 and grade 2 endometrioid carcinoma, and aggressive types were defined as grade 3 endometrioid carcinoma, serous carcinoma, clear cell carcinoma, carcinosarcoma, mucinous carcinoma, undifferentiated carcinoma, dedifferentiated carcinoma, and mixed carcinoma based on the 2023 FIGO criteria [25]. Additionally, staging was re‐evaluated or performed using the 2023 FIGO criteria [25].

**FIGURE 1:**
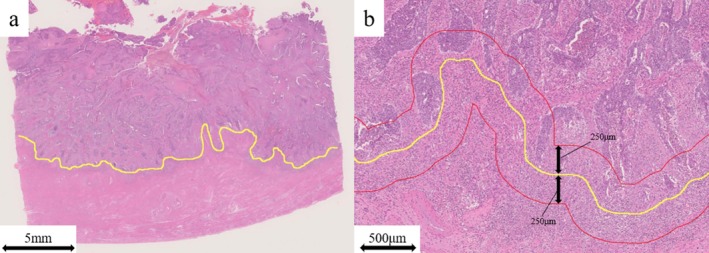
Representative images of the invasive frontline and the area for evaluating tumor‐infiltrating lymphocytes. (a) The yellow line indicates the manually annotated invasive frontline. (b) The area between two red lines automatically annotated by artificial intelligence indicates that within a width span of 500 μm, 250 μm toward both stroma and tumor from the invasive front line.

For all patients, hematoxylin and eosin (HE)‐stained slides were used for AI‐based pathological diagnosis. A single slide per case containing the invasive frontline of the tumor was selected and digitized using NanoZoomer SQ (Hamamatsu Photonics K.K., Shizuoka, Japan). Digital whole‐slide images were uploaded to the HALO software (Indica Labs, Corrales, NM, USA). On each uploaded slide, the invasive front line was manually annotated (Figure 1a). An area within a width span of 500 μm, extending 250 μm toward the stroma and tumor from the invasive frontline, was automatically annotated by AI (Figure 1b), and the area size (μm^2^) was automatically calculated.

**FIGURE 2:**
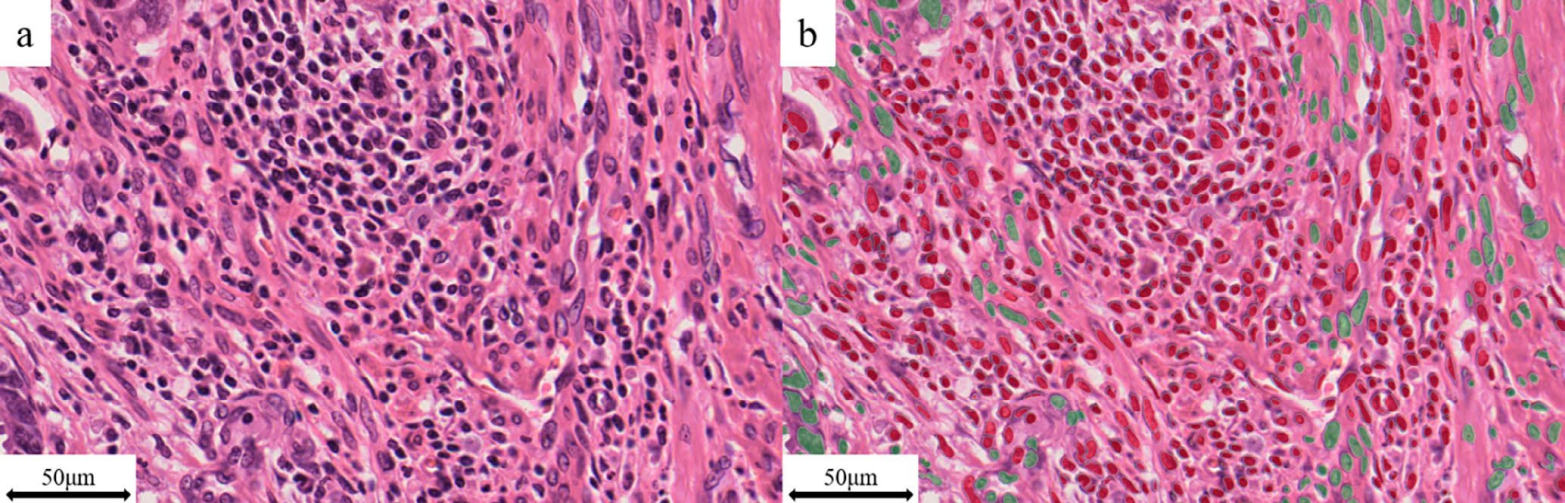
Representative images of lymphocytes (a) and structures detected by artificial intelligence (b). Red indicates lymphocytes, and green indicates other spherical structures, such as nuclei or histiocytes.

To evaluate lymphocytes, classifier training for lymphocytes was conducted using Object Phenotyper, which has the function of automatically detecting spherical structures, such as lymphocytes or histiocytes, in the designated area. From among the detected structures, only lymphocytes were manually selected, and these selected lymphocytes were then trained using AI. A total of 2235 lymphocytes from four cases were trained for 5025 iterations. The accuracy of the classifier was evaluated using the F1 scores for two cases that were not used for training. The F1 score explains the extent of agreement between automatic AI detection and manual annotation of lymphocytes. After training, the classifier was applied to all cases, and the number of lymphocytes in the AI‐annotated area was automatically counted. Representative images of the AI‐detected structures are shown in Figure 2, where red indicates lymphocytes and green indicates other spherical structures such as histiocytes. The average number of lymphocytes per area size (/μm^2^) was subsequently calculated for all cases, and the optimum cut‐off value was determined using the receiver operator characteristic (ROC) curve for recurrence or progression. Cases with lymphocyte counts per area equal to or greater than the cut‐off value were defined as the High‐TIL group, whereas those with counts less than the cut‐off value were defined as the Low‐TIL group.

In this study, the area for evaluating TILs was determined based on previous literature that reported the relationship between prognosis and TILs around the invasive frontline in ECs, and on exploratory analyses comparing multiple candidate areas [21]. Exploratory analyses included the following three candidate areas: (1) A range of 500 μm toward the stromal side from the invasive frontline, (2) A range of 250 μm toward the stromal side from the invasive frontline, (3) A total range of 500 μm, extending 250 μm toward the stroma and tumor from the invasive frontline. Details of these exploratory analyses are provided in Supporting Information (Figures S1 and S2 and Tables S1 and S2). Furthermore, to determine the cut‐off value for the High‐TIL and Low‐TIL groups, sensitivity analyses were performed using alternative cut‐off values, including the first quartile, median, and third quartile. The cut‐off value that showed the most appropriate prognostic association was selected for further analysis.

**FIGURE 3:**
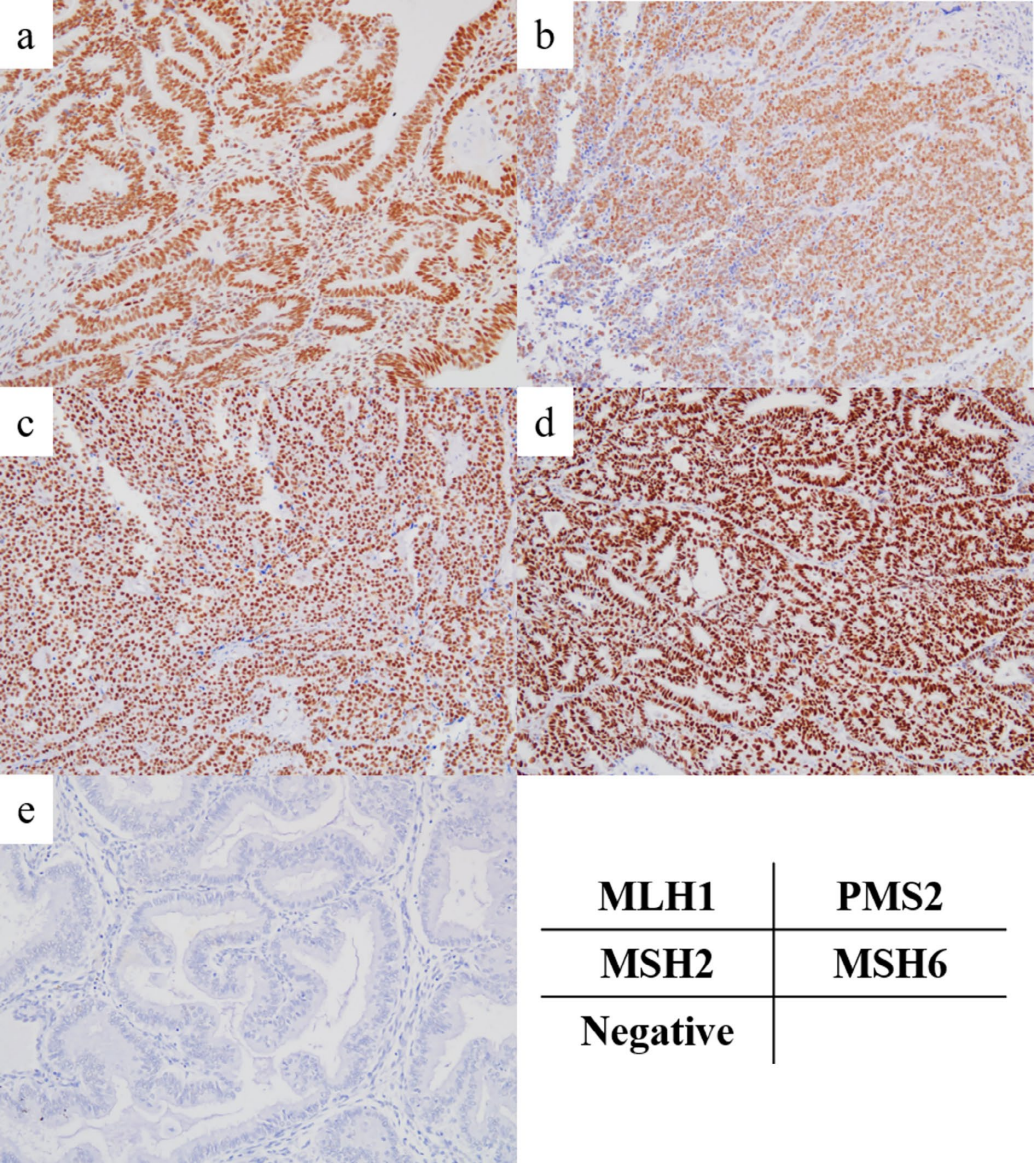
Representative images of positive immunohistochemistry (IHC) staining for mismatch repair proteins (×200) and complete absence of IHC staining. (a) MutL homolog 1 (MLH1). (b) Postmeiotic segregation increased 2 (PMS2). (c) MutS homolog 2 (MSH2). (d) MutS homolog 6 (MSH6). (e) Complete negative for staining.

Immunohistochemistry (IHC) was performed using formalin‐fixed, paraffin‐embedded tissues. Tissue microarray blocks were prepared as previously described [26]. For IHC staining, we used the following antibodies: mouse monoclonal antibody for mutL homolog 1 (MLH1) (clone ES05; dilution 1:50; Dako, Glostrup, Denmark); rabbit monoclonal antibody for postmeiotic segregation increased 2 (PMS2) (clone EP51; dilution 1:40; Dako); mouse monoclonal antibody for mutS homolog 2 (MSH2) (clone FE11; dilution 1:50; Dako); and rabbit monoclonal antibody for mutS homolog 6 (MSH6) (clone EP49; dilution 1:50; Dako). All slides were deparaffinized and rehydrated using a graded ethanol series. Endogenous peroxidase activity was blocked by adding methanol to 0.3% hydrogen peroxide. Antigen retrieval was performed using Tris/EDTA buffer (pH 9.0). Additionally, all the slides were boiled in Tris/EDTA buffer at 95°C for 20 min. Subsequently, the slides were incubated at room temperature for 20 min with primary antibodies against MLH1, MSH2, and MSH6, and for 30 min with primary antibodies against PMS2. In addition, the slides were incubated with the Dako EnVision+ system‐horseradish peroxidase‐labeled polymer as a secondary antibody for 30 min at room temperature. Specific antigen–antibody reactions were visualized with 0.2% diaminobenzidine tetrahydrochloride and hydrogen peroxide and counterstained with Mayer's hematoxylin. For each antibody, negative control experiments were performed without primary antibodies. Representative images of positive cells from each IHC staining are shown in Figure 3.

To interpret the IHC intensities of MLH1, PMS2, MSH2, and MSH6, the percentage of positive cells was scored as follows: (1) less than 1% of the nuclei stained; (2) 1%–33% of nuclei stained; (3) 34%–67% of the nuclei stained; and (4) > 67% of the nuclei stained. Staining intensity was scored as follows: 0, absent; 1, weak; 2, moderate; and 3, strong. The sum of these parameters was defined as the IHC score. Cases with less than 1% positive cells, regardless of intensity, were defined as negative (Figure 3e); cases with negative staining for one or more of the four proteins were defined as MMR‐deficient (dMMR); and others were defined as MMR‐proficient.

Statistical analyses were performed using JMP Pro 17 software (SAS Institute Inc., Cary, NC, USA). The chi‐square test, Fisher's exact test, and Mann–Whitney *U* test were used to evaluate the clinical significance of clinicopathological factors. Progression‐free survival (PFS) was defined as the period from the day of primary surgery to the day of death, disease recurrence, or progression. Overall survival (OS) was defined as the period from the day of the primary surgery to the day of death or the last follow‐up. PFS and OS curves were generated using the Kaplan–Meier method. The survival distribution was compared using a log‐rank test. Univariate and multivariate analyses were performed using Cox proportional hazard regression models. To evaluate whether the prognostic effect of TILs differed according to MMR status, TILs and MMR interaction terms were included in the Cox regression model. Variables with statistical significance in the univariate analysis were included in the multivariate analysis using backward stepwise selection. The level of statistical significance was set at *p* < 0.05.

In addition, subgroup analyses stratified by histological subtypes and MMR status were performed to evaluate the prognostic impact of TILs status.

## 3 Results

**FIGURE 4:**
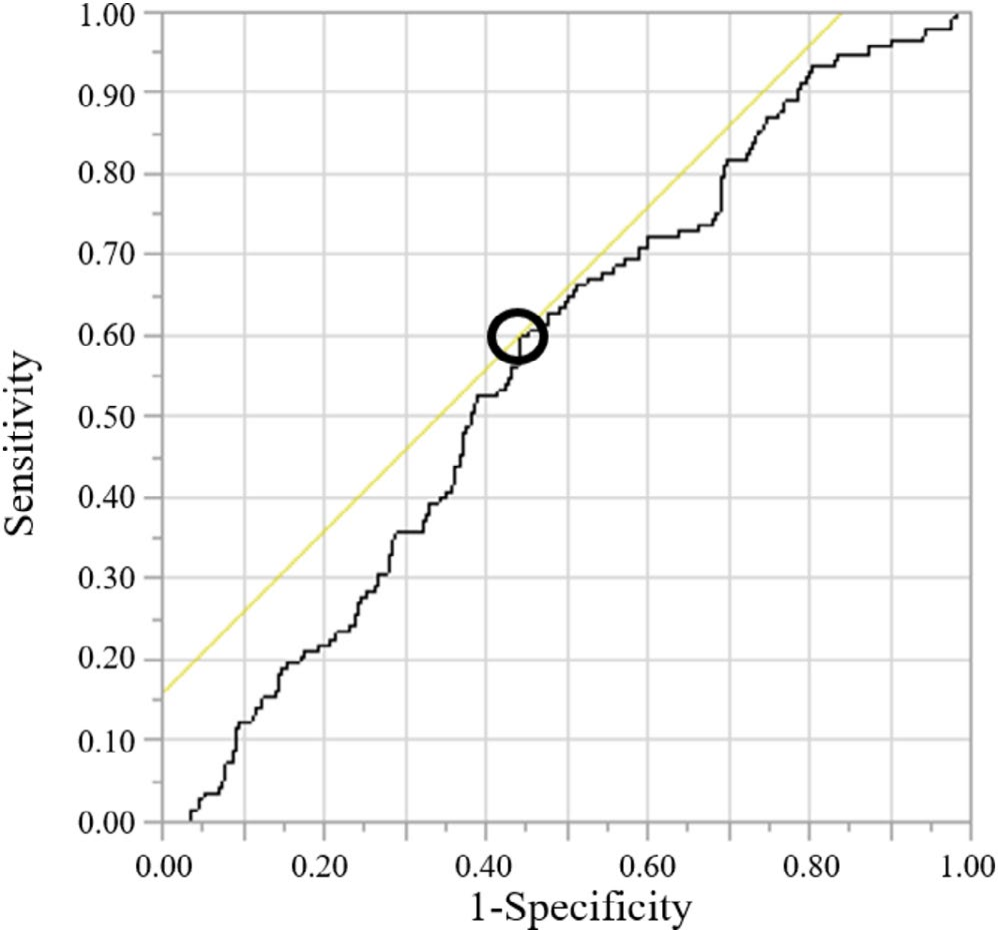
Receiver operating characteristics curve analysis of the average number of lymphocytes per area (/μm^2^) based on artificial intelligence for recurrence or progression. The area under the curve is 0.573.

A total of 659 patients with EC were included in this study. The F1 score was 0.81. The median number of lymphocytes counted by AI was 49 677 (21–242 372), and the median size of area for TIL evaluation was 10 893 771 (1 016 704–35 917 193) μm^2^. The median number of average lymphocytes per area size was 0.00493 (0.00001–0.01672)/μm^2^. The ROC curves for recurrence and progression are shown in Figure 4. The area under the curve was 0.570, and the cut‐off value was 0.00478. Sensitivity analyses using alternative cut‐off values, including the first quartile, median, and third quartile of TIL density, were performed. The cut‐off value defined by ROC analysis showed similar prognostic trends to the cut‐off value determined by median number, and the comparison of model fit using akaike information criteria and bayesian information criteria indicated similar results. Detailed results are provided in the Supporting Information (Figures S3 and S4 and Tables S3–S6).

**FIGURE 5:**
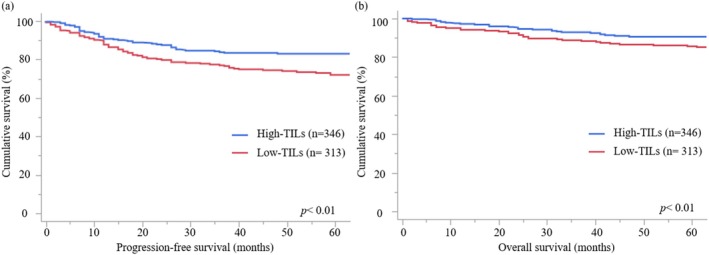
Survival analysis of the High‐TIL and Low‐TIL groups. (a) Progression‐free survival (PFS) curves of all cases in the High‐TIL and Low‐TIL groups. The High‐TIL group has a better prognosis than the Low‐TIL group (*p* < 0.01). (b) Overall survival (OS) curves of all cases in the High‐TIL and Low‐TIL groups. The High‐TIL group has a better prognosis than the Low‐TIL group (*p* < 0.01).

Of the 659 patients, 346 (52.5%) were assigned to the High‐TIL group and 313 (47.5%) were assigned to the Low‐TIL group, and all patients were included in the analysis (Table 1). The median observational period was 63 (1–394) months. Patients in the High‐TIL group were diagnosed at an earlier FIGO stage (*p* = 0.049), had a higher frequency of less than 50% of myometrial invasion (*p* = 0.02), and a more frequent dMMR status (*p* < 0.01) than those in the Low‐TIL group. Other factors did not differ significantly between the two groups. The High‐TIL group had better PFS (*p* < 0.01) and OS (*p* < 0.01) than the Low‐TIL group (Figure 5).

**TABLE 1:** Characteristics and results of immunohistochemical staining of 346 cases in the high‐tumor‐infiltrating lymphocyte group and 313 cases in the low‐tumor‐infiltrating lymphocyte group.

Variables	High‐TIL group		Low‐TIL group		*p*
	*n* = 346		*n* = 313		
Age (years) (%)	59.58 ± 10.47		60.86 ± 12.16		0.06
Average ± SD					0.06
≥ 60	157	(45.4)	165	(52.7)	
< 60	189	(54.6)	148	(47.3)	
Performance status (%)					0.40
0, 1	341	(98.6)	305	(97.4)	
≥ 2	5	(1.4)	8	(2.6)	
FIGO stage (%)					0.049
I	248	(71.7)	195	(62.3)	
II	27	(7.8)	40	(12.8)	
III	52	(15.0)	60	(19.2)	
IV	19	(5.5)	18	(5.7)	
Histology (%)					0.27
Grade 1 endometrioid carcinoma	166	(48.0)	165	(52.7)	
Grade 2 endometrioid carcinoma	76	(22.0)	51	(16.3)	
Grade 3 endometrioid carcinoma	36	(10.4)	36	(11.5)	
Serous carcinoma	16	(4.6)	15	(4.8)	
Clear cell carcinoma	9	(2.6)	9	(2.9)	
Carcinosarcoma	15	(4.3)	23	(7.4)	
Mucinous carcinoma	3	(0.9)	3	(0.9)	
Undifferentiated carcinoma	1	(0.3)	1	(0.3)	
Dedifferentiated carcinoma	1	(0.3)	1	(0.3)	
Mixed carcinoma	23	(6.6)	9	(2.9)	
Classified histological type (%)					0.80
Aggressive type	104	(30.1)	97	(31.0)	
Non‐aggressive type	242	(69.9)	216	(69.0)	
Myometrial invasion (%)					0.02
≥ 1/2	104	(30.1)	121	(38.7)	
< 1/2	242	(69.9)	192	(61.3)	
Cervical involvement (%)					0.06
Positive	40	(11.6)	53	(16.9)	
Negative	306	(88.4)	260	(83.1)	
Ovarian metastasis (%)					0.22
Positive	24	(6.9)	30	(9.6)	
Negative	322	(93.1)	283	(90.4)	
Lymph node metastasis (%)					0.91
Positive	43	(12.4)	40	(12.8)	
Negative	303	(87.6)	273	(87.2)	
Distant metastasis (%)					0.99
Positive	19	(5.5)	18	(5.8)	
Negative	327	(94.5)	295	(94.2)	
Lymphovascular invasion (%)					0.63
Positive	130	(37.6)	124	(39.6)	
Negative	216	(62.4)	189	(60.4)	
Ascites or lavage cytology (%)					0.21
Positive	41	(11.9)	48	(15.3)	
Negative	305	(88.1)	265	(84.7)	
Adjuvant therapy (%)					0.39
Yes	172	(49.7)	167	(53.4)	
No	174	(51.3)	146	(46.6)	
MMR status (%)					< 0.01
Deficient	147	(42.5)	101	(32.3)	
Proficient	199	(57.5)	212	(67.7)	

Abbreviations: FIGO, International Federation of Obstetrics and Gynecology; MMR, mismatch repair; SD, standard deviation; TIL, tumor‐infiltrating lymphocyte.

Multivariate analysis for PFS and OS revealed that TIL status was a prognostic factor for PFS (hazard ratio [HR] (95% confidence interval [CI]) 0.61 (0.43–0.87); *p* < 0.01) and OS (HR (95% CI) 0.54 (0.33–0.86); *p* = 0.01) (Table 2). We also performed subgroup analyses according to histological subtype and MMR status. In aggressive type, TIL status was a significant prognostic factor for PFS and OS, whereas in non‐aggressive types, multivariate analysis did not show the prognostic significance of TILs status (Figures S5 and S6 and Tables S7 and S8). In addition, in cases with dMMR, TIL status was a significant prognostic factor for both PFS and OS, whereas in cases with pMMR, TIL status was significantly associated with PFS but not with OS (Figures S7 and S8 and Tables S9 and S10). Detailed results, including ROC curves, Kaplan–Meier analyses, and Cox regression models for each subgroup, are shown in the Supporting Information.

**TABLE 2:** Univariate and multivariate analyses of progression‐free and overall survival rates for all cases.

Variables	Progression‐free survival						Overall survival					
	Univariate analysis			Multivariate analysis			Univariate analysis			Multivariate analysis		
	HR (95% CI)		*p*	HR (95% CI)		*p*	HR (95% CI)		*p*	HR (95% CI)		*p*
Age (years)												
≥ 60 vs. < 60	2.60	(1.81–3.75)	< 0.01	2.33	(1.58–3.44)	< 0.01	2.12	(1.35–3.34)	< 0.01	1.82	(1.12–2.96)	0.02
Performance status												
0, 1 vs. ≥ 2	0.29	(0.11–0.54)	< 0.01	0.53	(0.22–1.27)	0.15	0.18	(0.07–0.50)	< 0.01	0.73	(0.24–2.22)	0.57
Histological subtypes												
Aggressive type vs. Non‐aggressive type	4.24	(3.01–5.98)	< 0.01	2.25	(1.53–3.01)	< 0.01	4.32	(2.76–6.77)	< 0.01	2.51	(1.50–4.17)	< 0.01
Myometrial invasion												
< 1/2 vs. ≥ 1/2	0.24	(0.17–0.34)	< 0.01	0.61	(0.39–0.94)	0.03	0.25	(0.16–0.40)	< 0.01	0.46	(0.26–0.84)	0.01
Cervical involvement												
Positive vs. Negative	2.17	(1.47–3.24)	< 0.01	1.27	(0.83–1.94)	0.26	1.89	(1.12–3.19)	0.02	1.20	(0.67–2.12)	0.54
Ovarian metastasis												
Positive vs. Negative	5.75	(3.87–8.53)	< 0.01	1.77	(1.07–2.92)	0.03	8.49	(5.33–13.54)	< 0.01	2.82	(1.54–5.17)	< 0.01
Lymph node metastasis												
Positive vs. Negative	5.30	(3.73–7.53)	< 0.01	2.55	(1.66–3.93)	< 0.01	3.65	(2.29–5.83)	< 0.01	1.72	(0.97–3.08)	0.07
Distant metastasis												
Positive vs. Negative	13.12	(8.69–19.81)	< 0.01	6.91	(4.12–11.57)	< 0.01	13.56	(8.35–22.02)	< 0.01	6.76	(3.61–12.66)	< 0.01
Lymphovascular invasion												
Positive vs. Negative	4.15	(2.88–5.96)	< 0.01	2.16	(1.39–3.36)	< 0.01	3.28	(2.07–5.21)	< 0.01	1.85	(1.03–3.35)	0.04
Ascites or lavage cytology												
Positive vs. Negative	4.53	(3.17–6.45)	< 0.01	2.01	(1.27–3.18)	< 0.01	5.78	(3.72–8.97)	< 0.01	2.54	(1.38–4.69)	< 0.01
Adjuvant therapy												
Yes vs. No	2.37	(1.64–3.44)	< 0.01	0.46	(0.28–0.74)	< 0.01	1.91	(1.19–3.06)	< 0.01	0.26	(0.14–0.49)	< 0.01
TILs status												
High vs. Low	0.56	(0.40–0.79)	< 0.01	0.61	(0.43–0.87)	< 0.01	0.55	(0.35–0.85)	< 0.01	0.54	(0.33–0.86)	0.01
MMR status												
Deficiency vs. proficiency	1.21	(0.86–1.70)	0.26				1.42	(0.92–2.20)	0.11			
TILs and MMR interaction terms												
High‐TILs/MMR deficiency vs. others	0.84	(0.56–1.28)	0.42				0.83	(0.49–1.41)	0.49			

Abbreviations: CI, confidence interval; HR, hazard ratio; MMR, mismatch repair; TIL, tumor‐infiltrating lymphocyte.

## 4 Discussion

In the current study, we evaluated the AI‐based quantification of TILs in the area around the invasive frontline of EC and demonstrated that the High‐TIL group had better PFS and OS than the Low‐TIL group. Univariate and multivariate analyses revealed that TIL status was a prognostic factor for both PFS and OS.

Recently, the F1 score was used to assess the agreement between automatic detection using AI and manual annotation. The F1 score is the harmonic mean of precision (P), recall (R), and [2PR/(*P* + R)]. The value ranges between 0 and 1.0, and a value approaching 1.0 reflects high precision and recall [27]. In studies assessing TILs of malignant tumors, the F1 score has been used to determine the accuracy of the degree of agreement between lymphocytes detected automatically by AI and those manually annotated, yielding a score ranging between 0.78 and 0.97 [28, 29, 30]. In the current study, we also used the F1 score to evaluate the accuracy of the degree of agreement between automatic AI detection and manual annotation, resulting in an F1 score of 0.81. This value was within the range reported previously, indicating that its accuracy is retained.

In EC, TIL status was found to be associated with clinicopathological features, including FIGO stage, myometrial invasion, lymph node metastasis, distant metastasis, and lymphovascular invasion [15, 31, 32, 33, 34]. However, TILs were previously evaluated by human observers. In the current study, TILs were evaluated using AI, and patients in the High‐TIL group were diagnosed at an earlier FIGO stage and had a higher frequency of less than 50% of myometrial invasion than those in the Low‐TIL group. There was no discrepancy between the findings of previous studies and our study, underscoring the validity of AI‐based evaluation.

In addition, dMMR was identified in approximately 25%–30% of patients with EC and was associated with a high number of TILs [17, 35, 36]. In the current study, the rate of dMMR was higher than that reported previously, and dMMR was associated with TIL status. However, recent studies have shown that TIL status is associated with polymerase epsilon (POLE) status [37, 38]. Furthermore, several studies have reported the effect of POLE mutations on MMR function, as well as the possible co‐occurrence of dMMR and POLE mutations [39, 40, 41]. Additional studies are needed to investigate the relationship between TILs evaluated by AI, MMR status, and POLE status.

Nevertheless, the ideal prognostic model for evaluating any type of TIL in EC remains unclear. The clinical relevance of cluster of differentiation (CD) 4, CD8, and forkhead box P3 in EC has been reported [31, 32, 33]. Consistent with our study, previous studies have explored the clinical relevance of different TILs in EC [15, 31, 32, 33, 42]. Future studies should examine the clinical differences according to the type of AI‐detected TIL.

This study has some limitations. First, we evaluated TILs using HE slides, and orthogonal validation using other methods, such as deconvolution of bulk ribonucleic acid sequencing data or single‐cell and spatial transcriptomic analyses, was not performed. Second, this study was retrospective from a single institution, involving TIL evaluation. Third, the POLE status was not evaluated. Fourth, the deepest myometrial invasive frontline might not be the most appropriate site for evaluating TILs in cases with advanced‐stage, such as approaching the serosal surface or involving the cervix, lymph nodes, ovaries, and peritoneum. In addition, TILs at the metastatic sites or invasion frontline in the cervical invasive site were not assessed. Furthermore, the invasive frontline was manually annotated, and the inter‐observer reproducibility of this annotation process was not assessed. Those might represent a potential cause of inconsistency in the evaluation of TILs. Fifth, while this study demonstrated a high level of F1 score and the relationship between the TILs status and the MMR status, which indicated a high level of accuracy in the detection of lymphocytes, the dataset used to train the AI classifier was relatively small and did not consider the histological subtypes. Those might limit the model's consistency and ability. In addition, external validation using independent cohorts from other institutions was not performed. Furthermore, the analysis of features predicted by AI was limited to comparisons of clinicopathological characteristics, and other analyses considering tumor molecular profiles, key IHC markers, tumor mutation burden, or other data were not performed. Accordingly, additional studies are needed to compare HE and IHC staining for TIL evaluation, validate the observed findings through prospective studies from multiple institutions, evaluate the POLE status, conduct a more comprehensive evaluation of TILs across various stages, evaluate the consistency of invasive frontline annotation, include a large number of cases with considering the histological subtypes in training cohort, and perform analyses of features predicted by AI with molecular or genomic profiles. Furthermore, future studies should include external validation using independent cohorts and orthogonal validation using ribonucleic acid sequencing or single‐cell and spatial transcriptomic data to evaluate the performance of the AI‐based TILs assessment.

## 5 Conclusions

AI‐based evaluation of TILs in the area around the invasive frontline may be useful for predicting the prognosis of EC, and further studies are needed to establish methods for evaluating TILs in EC.

## Author Contributions

**Takahiro Einama:** formal analysis, resources, software, writing – review and editing. **Yuka Otsuka:** data curation, writing – review and editing. **Makiko Koga:** formal analysis, software. **Morikazu Miyamoto:** conceptualization, methodology, writing – original draft, project administration, visualization. **Hiroaki Soyama:** writing – review and editing. **Kohei Omatsu:** writing – review and editing. **Yoshinobu Hamada:** writing – review and editing. **Kento Kato:** data curation, writing – review and editing. **Kimiya Sato:** resources, supervision, writing – review and editing. **Masashi Takano:** project administration, supervision, writing – review and editing. **Taira Hada:** conceptualization, methodology, validation, formal analysis, investigation, data curation, writing – original draft, visualization. **Soichiro Kakimoto:** writing – review and editing. **Takanori Watanabe:** data curation, writing – review and editing. **Jin Suminokura:** investigation, writing – review and editing. **Tsubasa Ito:** investigation, writing – review and editing. **Naohisa Kishimoto:** writing – review and editing. Risa Tanabe: writing – review and editing. **Soko Nishimura:** writing – review and editing.

## Funding

The authors have nothing to report.

## Ethics Statement

This study was approved by the Institutional Review Board of National Defense Medical College, Tokorozawa, Japan. The records and information of all female participants included in the study were anonymized and de‐identified prior to analysis.

## Consent

The authors have nothing to report.

## Conflicts of Interest

The authors declare no conflicts of interest.

## Supporting information


**Table S1:** Univariate and Multivariate analysis of progression‐free survival and overall survival for all cases of endometrial carcinoma including tumor‐infiltrating lymphocytes evaluated using the area with the range of 500 μm toward the stromal side from the invasive frontline.
**Table S2:** Univariate and Multivariate analysis of progression‐free survival and overall survival for all cases of endometrial carcinoma including tumor‐infiltrating lymphocytes evaluated using the area with the range of 250 μm toward the stromal side from the invasive frontline.
**Table S3:** Univariate analysis of progression‐free survival and overall survival for all cases of endometrial carcinoma including the tumor‐infiltrative lymphocytes status classified based on the first quartile.
**Table S4:** Univariate and Multivariate analysis of progression‐free survival and overall survival for all cases of endometrial carcinoma including the tumor‐infiltrative lymphocytes status classified based on median number.
**Table S5:** Univariate and multivariate analysis of progression‐free survival and overall survival for all cases of endometrial carcinoma including the tumor‐infiltrative lymphocytes status classified based on the third quartile.
**Table S6:** Akaike and bayesian information criteria for multivariate analyses of progression‐free and overall survival according to tumor‐infiltrating lymphocyte status defined by different cut‐off values.
**Table S7:** Univariate and multivariate analysis of progression‐free survival and overall survival for cases of aggressive type endometrial carcinoma.
**Table S8:** Univariate and multivariate analysis of progression‐free survival and overall survival for cases of non‐aggressive type endometrial carcinoma.
**Table S9:** Univariate and multivariate analysis of progression‐free survival and overall survival for cases with mismatch repair deficiency.
**Table S10:** Univariate and multivariate analysis of progression‐free survival and overall survival for cases with mismatch repair proficiency.
**Figure S1:** Receiver operating characteristics (ROC) curve analysis of the average number of lymphocytes per area (/μm^2^) based on artificial intelligence for recurrence or progression. (a) ROC curve analysis of the average number of lymphocytes per area (/μm^2^) within the range of 500 μm toward the stromal side from the invasive frontline for recurrence or progression. The area under the curve is 0.570 and the cut‐off value was 0.00513. (b) ROC curve analysis of the average number of lymphocytes per area (/μm^2^) within the range of 250 μm toward the stromal side from the invasive frontline for recurrence or progression. The area under the curve is 0.573 and the cut‐off value was 0.00639.
**Figure S2:** Survival analysis of the High‐TIL and Low‐TIL groups. (a) Progression‐free survival (PFS) curves of all cases in the High‐TIL and Low‐TIL groups classified based on the area with the range of 500 μm toward the stromal side from the invasive frontline. The High‐TIL group has a better prognosis than the Low‐TIL group (*p* < 0.01). (b) Overall survival (OS) curves of all cases in the High‐TIL and Low‐TIL groups classified based on the area with the range of 500 μm toward the stromal side from the invasive frontline. The High‐TIL group has a better prognosis than the Low‐TIL group (*p* < 0.01). (c) PFS curves of all cases in the High‐TIL and Low‐TIL groups classified based on the area with the range of 250 μm toward the stromal side from the invasive frontline. The High‐TIL group has a better prognosis than the Low‐TIL group (*p* < 0.01). (d) OS curves of all cases in the High‐TIL and Low‐TIL groups classified based on the area with the range of 250 μm toward the stromal side from the invasive frontline. The High‐TIL group has a better prognosis than the Low‐TIL group (*p* < 0.01).
**Figure S3:** The lymphocytes density histogram. The first quartile, median, and third quartile numbers are 0.00301, 0.00493, and 0.00673, respectively.
**Figure S4:** Survival analysis of the High‐TIL and Low‐TIL groups. (a) Progression‐free survival (PFS) curves of all cases in the High‐TIL and Low‐TIL groups classified based on the first quartile number of the histogram of lymphocyte density. There is no prognostic significance between two groups (*p* = 0.48). (b) Overall survival (OS) curves of all cases in the High‐TIL and Low‐TIL groups classified based on the first quartile number of the histogram of lymphocyte density. There is no prognostic significance between two groups (*p* = 0.48). (c) PFS curves of all cases in the High‐TIL and Low‐TIL groups classified based on the median number of the histogram of lymphocyte density. The High‐TIL group has a better prognosis than the Low‐TIL group (*p* < 0.01). (d) OS curves of all cases in the High‐TIL and Low‐TIL groups classified based on the median number of the histogram of lymphocyte density. The High‐TIL group has a better prognosis than the Low‐TIL group (*p* < 0.01). (e) PFS curves of all cases in the High‐TIL and Low‐TIL groups classified based on the third quartile number of the histogram of lymphocyte density. The High‐TIL group has a better prognosis than the Low‐TIL group (*p* < 0.01). (f) OS curves of all cases in the High‐TIL and Low‐TIL groups classified based on the third quartile number of the histogram of lymphocyte density. The High‐TIL group has a better prognosis than the Low‐TIL group (*p* < 0.01).
**Figure S5:** Receiver operating characteristics (ROC) curve analysis of the average number of lymphocytes per area (/μm^2^) based on artificial intelligence for recurrence or progression. (a) ROC curve analysis of the average number of lymphocytes per area (/μm^2^) for recurrence or progression in cases with aggressive type endometrial carcinoma. The area under the curve is 0.614 and the cut‐off value was 0.00569. (b) ROC curve analysis of the average number of lymphocytes per area (/μm^2^) for recurrence or progression in cases with non‐aggressive type endometrial carcinoma. The area under the curve is 0.555 and the cut‐off value was 0.00478.
**Figure S6:** Survival analysis of the High‐TIL and Low‐TIL groups. (a) Progression‐free survival (PFS) curves of all cases in the High‐TIL and Low‐TIL groups in cases with aggressive type endometrial carcinoma. The High‐TIL group has a better prognosis than the Low‐TIL group (*p* < 0.01). (b) Overall survival (OS) curves of all cases in the High‐TIL and Low‐TIL groups in cases with aggressive type endometrial carcinoma. The High‐TIL group has a better prognosis than the Low‐TIL group (*p* < 0.01). (c) PFS curves of all cases in the High‐TIL and Low‐TIL groups in cases with non‐aggressive type endometrial carcinoma. The High‐TIL group has a better prognosis than the Low‐TIL group (*p* = 0.02). (d) OS curves of all cases in the High‐TIL and Low‐TIL groups in cases with non‐aggressive type endometrial carcinoma. There is no prognostic significance between two groups (*p* = 0.27).
**Figure S7:** Receiver operating characteristics (ROC) curve analysis of the average number of lymphocytes per area (/μm^2^) based on artificial intelligence for recurrence or progression. (a) ROC curve analysis of the average number of lymphocytes per area (/μm^2^) for recurrence or progression in cases with mismatch repair deficiency. The area under the curve is 0.597 and the cut‐off value was 0.00533. (b) ROC curve analysis of the average number of lymphocytes per area (/μm^2^) for recurrence or progression in cases with mismatch repair proficiency. The area under the curve is 0.558 and the cut‐off value was 0.00478.
**Figure S8:** Survival analysis of the High‐TIL and Low‐TIL groups. (a) Progression‐free survival (PFS) curves of all cases in the High‐TIL and Low‐TIL groups in cases with mismatch repair deficiency. The High‐TIL group has a better prognosis than the Low‐TIL group (*p* < 0.01). (b) Overall survival (OS) curves of all cases in the High‐TIL and Low‐TIL groups in cases with mismatch repair deficiency. The High‐TIL group has a better prognosis than the Low‐TIL group (*p* < 0.01). (c) PFS curves of all cases in the High‐TIL and Low‐TIL groups in cases with mismatch repair proficiency. The High‐TIL group has a better prognosis than the Low‐TIL group (*p* < 0.01). (d) OS curves of all cases in the High‐TIL and Low‐TIL groups in cases with mismatch repair proficiency. There is no prognostic significance between two groups (*p* = 0.06).

## Data Availability

The data that support the findings of this study are available on request from the corresponding author. The data are not publicly available due to privacy or ethical restrictions.
