# Supplementary material for: Establishment of a Model to Predict the Prognosis of Endometrial Carcinoma Using Tumor‐Infiltrating Lymphocytes Evaluated With Artificial Intelligence: A Retrospective Analysis
Source: Cancer Rep (Hoboken). 2026 Apr 9;9(4):e70535. doi: 10.1002/cnr2.70535 (PMC13066499; doi:10.1002/cnr2.70535)
Supplement: Supplementary file 1 — Table S1: Univariate and Multivariate analysis of progression‐free survival and overall survival for all cases of endometrial carcinoma including tumor‐infiltrating lymphocytes evaluated using the area with the range of 500 μm toward the stromal side from the invasive frontline. Table S2: Univariate and Multivariate analysis of progression‐free survival and overall survival for all cases of endometrial carcinoma including tumor‐infiltrating lymphocytes evaluated using the area with the range of 250 μm toward the stromal side from the invasive frontline. Table S3: Univariate analysis of progression‐free survival and overall survival for all cases of endometrial carcinoma including the tumor‐infiltrative lymphocytes status classified based on the first quartile. Table S4: Univariate and Multivariate analysis of progression‐free survival and overall survival for all cases of endometrial carcinoma including the tumor‐infiltrative lymphocytes status classified based on median number. Table S5: Univariate and multivariate analysis of progression‐free survival and overall survival for all cases of endometrial carcinoma including the tumor‐infiltrative lymphocytes status classified based on the third quartile. Table S6: Akaike and bayesian information criteria for multivariate analyses of progression‐free and overall survival according to tumor‐infiltrating lymphocyte status defined by different cut‐off values. Table S7: Univariate and multivariate analysis of progression‐free survival and overall survival for cases of aggressive type endometrial carcinoma. Table S8: Univariate and multivariate analysis of progression‐free survival and overall survival for cases of non‐aggressive type endometrial carcinoma. Table S9: Univariate and multivariate analysis of progression‐free survival and overall survival for cases with mismatch repair deficiency. Table S10: Univariate and multivariate analysis of progression‐free survival and overall survival for cases with mismatch repair pr [file CNR2-9-e70535-s001.docx]

**Table S1.** Univariate and Multivariate analysis of progression-free survival and overall survival for all cases of endometrial carcinoma including tumor-infiltrating lymphocytes evaluated using the area with the range of 500 µm toward the stromal side from the invasive frontline.

|  | Progression-free survival | | | | | | Overall survival | | | | | |
| --- | --- | --- | --- | --- | --- | --- | --- | --- | --- | --- | --- | --- |
|  | Univariate analysis | | | Multivariate analysis | | | Univariate analysis | | | Multivariate analysis | | |
| Variables | HR (95% CI) | | *p*-value | HR (95% CI) | | *p*-value | HR (95% CI) | | *p*-value | HR (95% CI) | | *p*-value |
| Age (years) |  |  |  |  |  |  |  |  |  |  |  |  |
| ≥60 vs. <60 | 2.60 | (1.81-3.75) | <0.01 | 2.36 | (1.60-3.48) | <0.01 | 2.12 | (1.35-3.34) | <0.01 | 1.79 | (1.10-2.93) | 0.02 |
| Performance status |  |  |  |  |  |  |  |  |  |  |  |  |
| 0, 1 vs. ≥2 | 0.29 | (0.11-0.54) | <0.01 | 0.46 | (0.19-1.09) | 0.08 | 0.18 | (0.07-0.50) | <0.01 | 0.65 | (0.21-1.94) | 0.44 |
| Histological subtypes |  |  |  |  |  |  |  |  |  |  |  |  |
| Aggressive type vs.  Non-aggressive type | 4.24 | (3.01-5.98) | <0.01 | 2.18 | (1.48-3.22) | <0.01 | 4.32 | (2.76-6.77) | <0.01 | 2.36 | (1.40-3.98) | <0.01 |
| Myometrial invasion |  |  |  |  |  |  |  |  |  |  |  |  |
| <1/2 vs. ≥1/2 | 0.24 | (0.17-0.34) | <0.01 | 0.58 | (0.37-0.90) | 0.02 | 0.25 | (0.16-0.40) | <0.01 | 0.45 | (0.24-0.81) | <0.01 |
| Cervical involvement |  |  |  |  |  |  |  |  |  |  |  |  |
| Positive vs. Negative | 2.17 | (1.47-3.24) | <0.01 | 1.32 | (0.87-2.01) | 0.20 | 1.89 | (1.12-3.19) | 0.02 | 1.26 | (0.72-2.22) | 0.42 |
| Ovarian metastasis |  |  |  |  |  |  |  |  |  |  |  |  |
| Positive vs. Negative | 5.75 | (3.87-8.53) | <0.01 | 1.76 | (1.05-2.94) | 0.03 | 8.49 | (5.33-13.54) | <0.01 | 2.82 | (1.52-5.21) | <0.01 |
| Lymph node metastasis |  |  |  |  |  |  |  |  |  |  |  |  |
| Positive vs. Negative | 5.30 | (3.73-7.53) | <0.01 | 2.43 | (1.58-3.74) | <0.01 | 3.65 | (2.29-5.83) | <0.01 | 1.60 | (0.91-2.81) | 0.10 |
| Distant metastasis |  |  |  |  |  |  |  |  |  |  |  |  |
| Positive vs. Negative | 13.12 | (8.69-19.81) | <0.01 | 6.52 | (3.84-11.06) | <0.01 | 13.56 | (8.35-22.02) | <0.01 | 5.97 | (3.19-11.17) | <0.01 |
| Lymphovascluar invasion |  |  |  |  |  |  |  |  |  |  |  |  |
| Positive vs. Negative | 4.15 | (2.88-5.96) | <0.01 | 2.09 | (1.34-3.26) | <0.01 | 3.28 | (2.07-5.21) | <0.01 | 1.74 | (0.96-3.16) | 0.07 |
| Ascites or lavage cytology |  |  |  |  |  |  |  |  |  |  |  |  |
| Positive vs. Negative | 4.53 | (3.17-6.45) | <0.01 | 1.94 | (1.23-3.08) | <0.01 | 5.78 | (3.72-8.97) | <0.01 | 2.63 | (1.43-4.84) | <0.01 |
| Adjuvant therapy |  |  |  |  |  |  |  |  |  |  |  |  |
| Yes vs. No | 2.37 | (1.64-3.44) | <0.01 | 0.48 | (0.30-0.76) | <0.01 | 1.91 | (1.19-3.06) | <0.01 | 0.27 | (0.14-0.50) | <0.01 |
| Tumor-infiltrating lymphocytes |  |  |  |  |  |  |  |  |  |  |  |  |
| High vs. Low | 0.53 | (0.37-0.76) | <0.01 | 0.77 | (0.53-1.12) | 0.17 | 0.44 | (0.27-0.72) | <0.01 | 0.55 | (0.39-0.92) | 0.02 |

Abbreviations: HR, hazard ratio; CI, confidence interval.

**Table S2.** Univariate and Multivariate analysis of progression-free survival and overall survival for all cases of endometrial carcinoma including tumor-infiltrating lymphocytes evaluated using the area with the range of 250 µm toward the stromal side from the invasive frontline.

|  | Progression-free survival | | | | | | Overall survival | | | | | |
| --- | --- | --- | --- | --- | --- | --- | --- | --- | --- | --- | --- | --- |
|  | Univariate analysis | | | Multivariate analysis | | | Univariate analysis | | | Multivariate analysis | | |
| Variables | HR (95% CI) | | *p*-value | HR (95% CI) | | *p*-value | HR (95% CI) | | *p*-value | HR (95% CI) | | *p*-value |
| Age (years) |  |  |  |  |  |  |  |  |  |  |  |  |
| ≥60 vs. <60 | 2.60 | (1.81-3.75) | <0.01 | 2.34 | (1.59-3.45) | <0.01 | 2.12 | (1.35-3.34) | <0.01 | 1.85 | (1.14-3.02) | 0.01 |
| Performance status |  |  |  |  |  |  |  |  |  |  |  |  |
| 0, 1 vs. ≥2 | 0.29 | (0.11-0.54) | <0.01 | 0.45 | (0.19-1.08) | 0.07 | 0.18 | (0.07-0.50) | <0.01 | 0.60 | (0.20-1.78) | 0.35 |
| Histological subtypes |  |  |  |  |  |  |  |  |  |  |  |  |
| Aggressive type vs.  Non-aggressive type | 4.24 | (3.01-5.98) | <0.01 | 2.20 | (1.50-3.23) | <0.01 | 4.32 | (2.76-6.77) | <0.01 | 2.34 | (1.39-3.93) | <0.01 |
| Myometrial invasion |  |  |  |  |  |  |  |  |  |  |  |  |
| <1/2 vs. ≥1/2 | 0.24 | (0.17-0.34) | <0.01 | 0.59 | (0.38-0.91) | 0.02 | 0.25 | (0.16-0.40) | <0.01 | 0.46 | (0.25-0.84) | 0.01 |
| Cervical involvement |  |  |  |  |  |  |  |  |  |  |  |  |
| Positive vs. Negative | 2.17 | (1.47-3.24) | <0.01 | 1.34 | (0.88-2.04) | 0.18 | 1.89 | (1.12-3.19) | 0.02 | 1.22 | (0.69-2.16) | 0.49 |
| Ovarian metastasis |  |  |  |  |  |  |  |  |  |  |  |  |
| Positive vs. Negative | 5.75 | (3.87-8.53) | <0.01 | 1.68 | (1.001-2.82) | 0.049 | 8.49 | (5.33-13.54) | <0.01 | 2.52 | (1.35-4.70) | <0.01 |
| Lymph node metastasis |  |  |  |  |  |  |  |  |  |  |  |  |
| Positive vs. Negative | 5.30 | (3.73-7.53) | <0.01 | 2.44 | (1.59-3.75) | <0.01 | 3.65 | (2.29-5.83) | <0.01 | 1.61 | (0.91-2.86) | 0.10 |
| Distant metastasis |  |  |  |  |  |  |  |  |  |  |  |  |
| Positive vs. Negative | 13.12 | (8.69-19.81) | <0.01 | 6.74 | (4.00-11.36) | <0.01 | 13.56 | (8.35-22.02) | <0.01 | 6.53 | (3.46-12.32) | <0.01 |
| Lymphovascluar invasion |  |  |  |  |  |  |  |  |  |  |  |  |
| Positive vs. Negative | 4.15 | (2.88-5.96) | <0.01 | 2.06 | (1.32-3.23) | <0.01 | 3.28 | (2.07-5.21) | <0.01 | 1.71 | (0.94-3.13) | 0.08 |
| Ascites or lavage cytology |  |  |  |  |  |  |  |  |  |  |  |  |
| Positive vs. Negative | 4.53 | (3.17-6.45) | <0.01 | 1.93 | (1.22-3.05) | <0.01 | 5.78 | (3.72-8.97) | <0.01 | 2.55 | (1.38-4.73) | <0.01 |
| Adjuvant therapy |  |  |  |  |  |  |  |  |  |  |  |  |
| Yes vs. No | 2.37 | (1.64-3.44) | <0.01 | 0.45 | (0.30-0.76) | <0.01 | 1.91 | (1.19-3.06) | <0.01 | 0.28 | (0.15-0.51) | <0.01 |
| Tumor-infiltrating lymphocytes |  |  |  |  |  |  |  |  |  |  |  |  |
| High vs. Low | 0.44 | (0.29-0.69) | <0.01 | 0.67 | (0.43-1.05) | 0.08 | 0.35 | (0.19-0.63) | <0.01 | 0.48 | (0.26-0.91) | 0.02 |

Abbreviations: HR, hazard ratio; CI, confidence interval.

**Table S3.** Univariate analysis of progression-free survival and overall survival for all cases of endometrial carcinoma including the tumor-infiltrative lymphocytes status classified based on the first quartile.

|  | Progression-free survival | | | | | | Overall survival | | | | | |
| --- | --- | --- | --- | --- | --- | --- | --- | --- | --- | --- | --- | --- |
|  | Univariate analysis | | | Multivariate analysis | | | Univariate analysis | | | Multivariate analysis | | |
| Variables | HR (95% CI) | | *p*-Value | HR (95% CI) | | *p*-Value | HR (95% CI) | | *p*-Value | HR (95% CI) | | *p*-Value |
| Age (years) |  |  |  |  |  |  |  |  |  |  |  |  |
| ≥60 vs. <60 | 2.60 | (1.81-3.75) | <0.01 | 2.89 | (1.62-3.52) | <0.01 | 2.12 | (1.35-3.34) | <0.01 | 1.87 | (1.15-3.05) | 0.01 |
| Performance status |  |  |  |  |  |  |  |  |  |  |  |  |
| 0, 1 vs. ≥2 | 0.29 | (0.11-0.54) | <0.01 | 0.44 | (0.18-1.04) | 0.06 | 0.18 | (0.07-0.50) | <0.01 | 0.56 | (0.19-1.66) | 0.29 |
| Histological subtypes |  |  |  |  |  |  |  |  |  |  |  |  |
| Aggressive type vs.  Non-aggressive type | 4.24 | (3.01-5.98) | <0.01 | 2.27 | (1.54-3.33) | <0.01 | 4.32 | (2.76-6.77) | <0.01 | 2.70 | (1.62-4.49) | <0.01 |
| Myometrial invasion |  |  |  |  |  |  |  |  |  |  |  |  |
| <1/2 vs. ≥1/2 | 0.24 | (0.17-0.34) | <0.01 | 0.56 | (0.36-0.88) | 0.01 | 0.25 | (0.16-0.40) | <0.01 | 0.44 | (0.25-0.82) | <0.01 |
| Cervical involvement |  |  |  |  |  |  |  |  |  |  |  |  |
| Positive vs. Negative | 2.17 | (1.47-3.24) | <0.01 | 1.33 | (0.87-2.02) | 0.19 | 1.89 | (1.12-3.19) | 0.02 | 1.19 | (0.67-2.11) | 0.55 |
| Ovarian metastasis |  |  |  |  |  |  |  |  |  |  |  |  |
| Positive vs. Negative | 5.75 | (3.87-8.53) | <0.01 | 1.76 | (1.05-2.95) | 0.03 | 8.49 | (5.33-13.54) | <0.01 | 2.81 | (1.51-5.24) | <0.01 |
| Lymph node metastasis |  |  |  |  |  |  |  |  |  |  |  |  |
| Positive vs. Negative | 5.30 | (3.73-7.53) | <0.01 | 2.35 | (1.53-3.62) | <0.01 | 3.65 | (2.29-5.83) | <0.01 | 1.51 | (0.85-2.66) | 0.16 |
| Distant metastasis |  |  |  |  |  |  |  |  |  |  |  |  |
| Positive vs. Negative | 13.12 | (8.69-19.81) | <0.01 | 6.84 | (4.05-11.56) | <0.01 | 13.56 | (8.35-22.02) | <0.01 | 5.95 | (3.16-11.21) | <0.01 |
| Lymphovascluar invasion |  |  |  |  |  |  |  |  |  |  |  |  |
| Positive vs. Negative | 4.15 | (2.88-5.96) | <0.01 | 2.11 | (1.36-3.29) | <0.01 | 3.28 | (2.07-5.21) | <0.01 | 1.82 | (1.01-3.28) | 0.045 |
| Ascites or lavage cytology |  |  |  |  |  |  |  |  |  |  |  |  |
| Positive vs. Negative | 4.53 | (3.17-6.45) | <0.01 | 1.91 | (1.20-3.03) | <0.01 | 5.78 | (3.72-8.97) | <0.01 | 2.42 | (1.30-4.50) | <0.01 |
| Adjuvant therapy |  |  |  |  |  |  |  |  |  |  |  |  |
| Yes vs. No | 2.37 | (1.64-3.44) | <0.01 | 0.47 | (0.30-0.76) | <0.01 | 1.91 | (1.19-3.06) | <0.01 | 0.28 | (0.15-0.51) | <0.01 |
| Tumor-infiltrating lymphocytes |  |  |  |  |  |  |  |  |  |  |  |  |
| High vs. Low | 0.87 | (0.60-1.27) | 0.48 |  |  |  | 1.04 | (0.62-1.74) | 0.88 |  |  |  |

Abbreviations: HR, hazard ratio; CI, confidence interval.

**Table S4.** Univariate and Multivariate analysis of progression-free survival and overall survival for all cases of endometrial carcinoma including the tumor-infiltrative lymphocytes status classified based on median number.

|  | Progression-free survival | | | | | | Overall survival | | | | | |
| --- | --- | --- | --- | --- | --- | --- | --- | --- | --- | --- | --- | --- |
|  | Univariate analysis | | | Multivariate analysis | | | Univariate analysis | | | Multivariate analysis | | |
| Variables | HR (95% CI) | | *p*-value | HR (95% CI) | | *p*-value | HR (95% CI) | | *p*-value | HR (95% CI) | | *p*-value |
| Age (years) |  |  |  |  |  |  |  |  |  |  |  |  |
| ≥60 vs. <60 | 2.60 | (1.81-3.75) | <0.01 | 2.32 | (1.58-3.42) | <0.01 | 2.12 | (1.35-3.34) | <0.01 | 1.80 | (1.11-2.94) | 0.02 |
| Performance status |  |  |  |  |  |  |  |  |  |  |  |  |
| 0, 1 vs. ≥2 | 0.29 | (0.11-0.54) | <0.01 | 0.52 | (0.22-1.25) | 0.14 | 0.18 | (0.07-0.50) | <0.01 | 0.73 | (0.24-2.26) | 0.60 |
| Histological subtypes |  |  |  |  |  |  |  |  |  |  |  |  |
| Aggressive type vs.  Non-aggressive type | 4.24 | (3.01-5.98) | <0.01 | 2.29 | (1.56-3.36) | <0.01 | 4.32 | (2.76-6.77) | <0.01 | 2.53 | (1.52-4.21) | <0.01 |
| Myometrial invasion |  |  |  |  |  |  |  |  |  |  |  |  |
| <1/2 vs. ≥1/2 | 0.24 | (0.17-0.34) | <0.01 | 0.61 | (0.39-0.95) | 0.03 | 0.25 | (0.16-0.40) | <0.01 | 0.47 | (0.26-0.85) | 0.01 |
| Cervical involvement |  |  |  |  |  |  |  |  |  |  |  |  |
| Positive vs. Negative | 2.17 | (1.47-3.24) | <0.01 | 1.29 | (0.84-1.96) | 0.24 | 1.89 | (1.12-3.19) | 0.02 | 1.21 | (0.68-2.14) | 0.52 |
| Ovarian metastasis |  |  |  |  |  |  |  |  |  |  |  |  |
| Positive vs. Negative | 5.75 | (3.87-8.53) | <0.01 | 1.79 | (1.08-2.98) | 0.02 | 8.49 | (5.33-13.54) | <0.01 | 2.87 | (1.57-5.26) | <0.01 |
| Lymph node metastasis |  |  |  |  |  |  |  |  |  |  |  |  |
| Positive vs. Negative | 5.30 | (3.73-7.53) | <0.01 | 2.55 | (1.65-3.94) | <0.01 | 3.65 | (2.29-5.83) | <0.01 | 1.75 | (0.98-3.14) | 0.06 |
| Distant metastasis |  |  |  |  |  |  |  |  |  |  |  |  |
| Positive vs. Negative | 13.12 | (8.69-19.81) | <0.01 | 6.91 | (4.12-11.61) | <0.01 | 13.56 | (8.35-22.02) | <0.01 | 6.86 | (3.65-12.86) | <0.01 |
| Lymphovascluar invasion |  |  |  |  |  |  |  |  |  |  |  |  |
| Positive vs. Negative | 4.15 | (2.88-5.96) | <0.01 | 2.16 | (1.38-3.36) | <0.01 | 3.28 | (2.07-5.21) | <0.01 | 1.85 | (1.02-3.34) | 0.04 |
| Ascites or lavage cytology |  |  |  |  |  |  |  |  |  |  |  |  |
| Positive vs. Negative | 4.53 | (3.17-6.45) | <0.01 | 1.98 | (1.25-3.14) | <0.01 | 5.78 | (3.72-8.97) | <0.01 | 2.55 | (0.14-0.48) | <0.01 |
| Adjuvant therapy |  |  |  |  |  |  |  |  |  |  |  |  |
| Yes vs. No | 2.37 | (1.64-3.44) | <0.01 | 0.45 | (0.28-0.73) | <0.01 | 1.91 | (1.19-3.06) | <0.01 | 0.26 | (0.14-0.48) | <0.01 |
| Tumor-infiltrating lymphocytes |  |  |  |  |  |  |  |  |  |  |  |  |
| High vs. Low | 0.59 | (0.42-0.83) | <0.01 | 0.63 | (0.44-0.91) | 0.01 | 0.54 | (0.35-0.85) | <0.01 | 0.51 | (0.32-0.83) | <0.01 |

Abbreviations: HR, hazard ratio; CI, confidence interval.

**Table S5.** Univariate and multivariate analysis of progression-free survival and overall survival for all cases of endometrial carcinoma including the tumor-infiltrative lymphocytes status classified based on the third quartile.

|  | Progression-free survival | | | | | | Overall survival | | | | | |
| --- | --- | --- | --- | --- | --- | --- | --- | --- | --- | --- | --- | --- |
|  | Univariate analysis | | | Multivariate analysis | | | Univariate analysis | | | Multivariate analysis | | |
| Variables | HR (95% CI) | | *p*-Value | HR (95% CI) | | *p*-Value | HR (95% CI) | | *p*-Value | HR (95% CI) | | *p*-Value |
| Age (years) |  |  |  |  |  |  |  |  |  |  |  |  |
| ≥60 vs. <60 | 2.60 | (1.81-3.75) | <0.01 | 2.34 | (1.59-3.45) | <0.01 | 2.12 | (1.35-3.34) | <0.01 | 1.82 | (1.11-2.97) | 0.02 |
| Performance status |  |  |  |  |  |  |  |  |  |  |  |  |
| 0, 1 vs. ≥2 | 0.29 | (0.11-0.54) | <0.01 | 0.44 | (0.19-1.06) | 0.07 | 0.18 | (0.07-0.50) | <0.01 | 0.57 | (0.19-1.69) | 0.31 |
| Histological subtypes |  |  |  |  |  |  |  |  |  |  |  |  |
| Aggressive type vs.  Non-aggressive type | 4.24 | (3.01-5.98) | <0.01 | 2.32 | (1.58-3.40) | <0.01 | 4.32 | (2.76-6.77) | <0.01 | 2.72 | (1.64-4.52) | <0.01 |
| Myometrial invasion |  |  |  |  |  |  |  |  |  |  |  |  |
| <1/2 vs. ≥1/2 | 0.24 | (0.17-0.34) | <0.01 | 0.59 | (0.38-0.93) | 0.02 | 0.25 | (0.16-0.40) | <0.01 | 0.47 | (0.26-0.87) | 0.02 |
| Cervical involvement |  |  |  |  |  |  |  |  |  |  |  |  |
| Positive vs. Negative | 2.17 | (1.47-3.24) | <0.01 | 1.36 | (0.89-2.07) | 0.15 | 1.89 | (1.12-3.19) | 0.02 | 1.22 | (0.69-2.15) | 0.50 |
| Ovarian metastasis |  |  |  |  |  |  |  |  |  |  |  |  |
| Positive vs. Negative | 5.75 | (3.87-8.53) | <0.01 | 1.68 | (1.003-2.81) | 0.048 | 8.49 | (5.33-13.54) | <0.01 | 2.65 | (1.42-4.93) | <0.01 |
| Lymph node metastasis |  |  |  |  |  |  |  |  |  |  |  |  |
| Positive vs. Negative | 5.30 | (3.73-7.53) | <0.01 | 2.47 | (1.60-3.81) | <0.01 | 3.65 | (2.29-5.83) | <0.01 | 1.60 | (0.90-2.85) | 0.11 |
| Distant metastasis |  |  |  |  |  |  |  |  |  |  |  |  |
| Positive vs. Negative | 13.12 | (8.69-19.81) | <0.01 | 6.71 | (3.98-11.32) | <0.01 | 13.56 | (8.35-22.02) | <0.01 | 5.85 | (3.11-10.97) | <0.01 |
| Lymphovascluar invasion |  |  |  |  |  |  |  |  |  |  |  |  |
| Positive vs. Negative | 4.15 | (2.88-5.96) | <0.01 | 2.08 | (1.33-3.23) | <0.01 | 3.28 | (2.07-5.21) | <0.01 | 1.80 | (1.001-3.23) | 0.049 |
| Ascites or lavage cytology |  |  |  |  |  |  |  |  |  |  |  |  |
| Positive vs. Negative | 4.53 | (3.17-6.45) | <0.01 | 1.92 | (1.21-3.05) | <0.01 | 5.78 | (3.72-8.97) | <0.01 | 2.39 | (1.28-4.46) | <0.01 |
| Adjuvant therapy |  |  |  |  |  |  |  |  |  |  |  |  |
| Yes vs. No | 2.37 | (1.64-3.44) | <0.01 | 0.47 | (0.29-0.76) | <0.01 | 1.91 | (1.19-3.06) | <0.01 | 0.27 | (0.15-0.51) | <0.01 |
| Tumor-infiltrating lymphocytes |  |  |  |  |  |  |  |  |  |  |  |  |
| High vs. Low | 0.55 | (0.35-0.86) | <0.01 | 0.75 | (0.46-1.21) | 0.23 | 0.41 | (0.21-0.77) | <0.01 | 0.67 | (0.34-1.32) | 0.25 |

Abbreviations: HR, hazard ratio; CI, confidence interval.

**Table S6.** Akaike and bayesian information criteria for multivariate analyses of progression-free and overall survival according to tumor-infiltrating lymphocyte status defined by different cut-off values.

|  | Progression-free survival | | Overall survival | |
| --- | --- | --- | --- | --- |
|  | AIC | BIC | AIC | BIC |
| Cutoff value defined by ROC analysis | 1445.34 | 1498.75 | 824.55 | 877.96 |
| Cutoff value defined by median number | 1446.34 | 1499.75 | 823.57 | 877.98 |

Abbreviations: AIC, akaike information criteria; BIC, bayesian information criteria; ROC, receiver operating characteristic.

**Table S7.** Univariate and multivariate analysis of progression-free survival and overall survival for cases of aggressive type endometrial carcinoma.

|  | Progression-free survival | | | | | | Overall survival | | | | | |
| --- | --- | --- | --- | --- | --- | --- | --- | --- | --- | --- | --- | --- |
|  | Univariate analysis | | | Multivariate analysis | | | Univariate analysis | | | Multivariate analysis | | |
| Variables | HR (95% CI) | | *p*-Value | HR (95% CI) | | *p*-Value | HR (95% CI) | | *p*-Value | HR (95% CI) | | *p*-Value |
| Age (years) |  |  |  |  |  |  |  |  |  |  |  |  |
| ≥60 vs. <60 | 1.75 | (1.09-2.83) | 0.02 | 2.52 | (1.50-4.22) | <0.01 | 1.43 | (0.80-2.58) | 0.23 |  |  |  |
| Performance status |  |  |  |  |  |  |  |  |  |  |  |  |
| 0, 1 vs. ≥2 | 0.69 | (0.22-2.19) | 0.53 |  |  |  | 0.60 | (0.15-2.47) | 0.48 |  |  |  |
| Myometrial invasion |  |  |  |  |  |  |  |  |  |  |  |  |
| <1/2 vs. ≥1/2 | 0.40 | (0.25-0.64) | <0.01 | 0.90 | (0.53-1.53) | 0.69 | 0.36 | (0.20-0.66) | <0.01 | 0.63 | (0.31-1.29) | 0.21 |
| Cervical involvement |  |  |  |  |  |  |  |  |  |  |  |  |
| Positive vs. Negative | 1.38 | (0.84-2.30) | 0.21 |  |  |  | 1.46 | (0.78-2.76) | 0.24 |  |  |  |
| Ovarian metastasis |  |  |  |  |  |  |  |  |  |  |  |  |
| Positive vs. Negative | 3.27 | (1.98-5.40) | <0.01 | 1.10 | (0.61-2.00) | 0.75 | 4.69 | (2.59-8.50) | <0.01 | 1.85 | (0.93-3.66) | 0.08 |
| Lymph node metastasis |  |  |  |  |  |  |  |  |  |  |  |  |
| Positive vs. Negative | 3.61 | (2.32-5.63) | <0.01 | 2.51 | (1.51-4.18) | <0.01 | 2.49 | (1.42-4.37) | <0.01 | 1.84 | (0.98-3.46) | 0.06 |
| Distant metastasis |  |  |  |  |  |  |  |  |  |  |  |  |
| Positive vs. Negative | 8.22 | (4.96-13.61) | <0.01 | 7.65 | (4.17-14.00) | <0.01 | 8.04 | (4.46-14.50) | <0.01 | 5.02 | (2.59-9.74) | <0.01 |
| Lymphovascluar invasion |  |  |  |  |  |  |  |  |  |  |  |  |
| Positive vs. Negative | 2.96 | (1.75-5.01) | <0.01 | 2.24 | (1.21-4.11) | <0.01 | 2.68 | (1.37-5.23) | <0.01 | 1.54 | (0.69-3.46) | 0.30 |
| Ascites or lavage cytology |  |  |  |  |  |  |  |  |  |  |  |  |
| Positive vs. Negative | 3.26 | (2.08-5.10) | <0.01 | 1.67 | (0.98-2.85) | 0.06 | 4.06 | (2.31-7.13) | <0.01 | 1.69 | (0.86-3.31) | 0.13 |
| Adjuvant therapy |  |  |  |  |  |  |  |  |  |  |  |  |
| Yes vs. No | 0.96 | (0.55-1.67) | 0.87 |  |  |  | 0.71 | (0.36-1.38) | 0.31 |  |  |  |
| Tumor-infiltrating lymphocytes |  |  |  |  |  |  |  |  |  |  |  |  |
| High vs. Low | 0.46 | (0.28-0.76) | <0.01 | 0.49 | (0.29-0.83) | <0.01 | 0.34 | (0.17-0.69) | <0.01 | 0.41 | (0.20-0.87) | 0.02 |

Abbreviations: HR, hazard ratio; CI, confidence interval.

**Table S8.** Univariate and multivariate analysis of progression-free survival and overall survival for cases of non-aggressive type endometrial carcinoma.

|  | Progression-free survival | | | | | | Overall survival | | | | | |
| --- | --- | --- | --- | --- | --- | --- | --- | --- | --- | --- | --- | --- |
|  | Univariate analysis | | | Multivariate analysis | | | Univariate analysis | | | Multivariate analysis | | |
| Variables | HR (95% CI) | | *p*-Value | HR (95% CI) | | *p*-Value | HR (95% CI) | | *p*-Value | HR (95% CI) | | *p*-Value |
| Age (years) |  |  |  |  |  |  |  |  |  |  |  |  |
| ≥60 vs. <60 | 2.86 | (1.64-5.01) | <0.01 | 2.21 | (1.18-4.16) | 0.01 | 2.28 | (1.11-4.68) | 0.02 | 2.36 | (1.03-5.40) | 0.04 |
| Performance status |  |  |  |  |  |  |  |  |  |  |  |  |
| 0, 1 vs. ≥2 | 0.07 | (0.02-0.24) | <0.01 | 0.15 | (0.04-0.56) | <0.01 | 0.03 | (0.01-0.16) | <0.01 | 0.05 | (0.01-0.32) | <0.01 |
| Myometrial invasion |  |  |  |  |  |  |  |  |  |  |  |  |
| <1/2 vs. ≥1/2 | 0.22 | (0.13-0.38) | <0.01 | 0.27 | (0.13-0.59) | <0.01 | 0.28 | (0.14-0.56) | <0.01 | 0.39 | (0.16-0.95) | 0.04 |
| Cervical involvement |  |  |  |  |  |  |  |  |  |  |  |  |
| Positive vs. Negative | 2.51 | (1.33-4.76) | <0.01 | 1.17 | (0.54-2.54) | 0.69 | 1.57 | (0.61-4.10) | 0.35 |  |  |  |
| Ovarian metastasis |  |  |  |  |  |  |  |  |  |  |  |  |
| Positive vs. Negative | 7.27 | (3.82-13.83) | <0.01 | 4.25 | (1.75-10.32) | <0.01 | 11.26 | (5.26-24.09) | <0.01 | 5.78 | (1.81-18.42) | <0.01 |
| Lymph node metastasis |  |  |  |  |  |  |  |  |  |  |  |  |
| Positive vs. Negative | 4.22 | (2.26-7.86) | <0.01 | 2.47 | (1.08-5.66) | 0.03 | 2.31 | (0.89-6.02) | 0.09 |  |  |  |
| Distant metastasis |  |  |  |  |  |  |  |  |  |  |  |  |
| Positive vs. Negative | 9.54 | (4.05-22.47) | <0.01 | 6.69 | (2.21-20.20) | <0.01 | 10.93 | (4.15-28.79) | <0.01 | 2.35 | (0.67-8.25) | 0.18 |
| Lymphovascluar invasion |  |  |  |  |  |  |  |  |  |  |  |  |
| Positive vs. Negative | 3.18 | (1.87-5.41) | <0.01 | 1.39 | (0.69-2.80) | 0.36 | 2.04 | (1.02-4.10) | 0.045 | 1.18 | (0.48-2.89) | 0.72 |
| Ascites or lavage cytology |  |  |  |  |  |  |  |  |  |  |  |  |
| Positive vs. Negative | 3.78 | (2.06-6.93) | <0.01 | 1.89 | (0.77-4.62) | 0.16 | 5.21 | (2.49-10.89) | <0.01 | 1.90 | (0.64-5.67) | 0.25 |
| Adjuvant therapy |  |  |  |  |  |  |  |  |  |  |  |  |
| Yes vs. No | 1.87 | (1.10-3.18) | 0.02 | 0.39 | (0.18-0.81) | 0.01 | 1.57 | (0.77-3.17) | 0.21 |  |  |  |
| Tumor-infiltrating lymphocytes |  |  |  |  |  |  |  |  |  |  |  |  |
| High vs. Low | 0.52 | (0.30-0.89) | 0.02 | 0.60 | (0.34-1.05) | 0.07 | 0.68 | (0.34-1.36) | 0.28 |  |  |  |

Abbreviations: HR, hazard ratio; CI, confidence interval.

**Table S9.** Univariate and multivariate analysis of progression-free survival and overall survival for cases with mismatch repair deficiency.

|  | Progression-free survival | | | | | | Overall survival | | | | | |
| --- | --- | --- | --- | --- | --- | --- | --- | --- | --- | --- | --- | --- |
|  | Univariate analysis | | | Multivariate analysis | | | Univariate analysis | | | Multivariate analysis | | |
| Variables | HR (95% CI) | | *p*-value | HR (95% CI) | | *p*-value | HR (95% CI) | | *p*-value | HR (95% CI) | | *p*-value |
| Age (years) |  |  |  |  |  |  |  |  |  |  |  |  |
| ≥60 vs. <60 | 2.12 | (1.24-3.62) | <0.01 | 2.21 | (1.23-3.95) | <0.01 | 1.62 | (0.86-3.05) | 0.13 |  |  |  |
| Performance status |  |  |  |  |  |  |  |  |  |  |  |  |
| 0, 1 vs. ≥2 | 0.39 | (0.12-1.24) | 0.11 |  |  |  | 0.22 | (0.07-0.72) | 0.01 | 1.20 | (0.30-4.80) | 0.79 |
| Histological subtypes |  |  |  |  |  |  |  |  |  |  |  |  |
| Aggressive type vs.  Non-aggressive type | 3.32 | (1.95-5.64) | <0.01 | 1.43 | (0.75-2.72) | 0.28 | 2.81 | (1.49-5.29) | <0.01 | 1.52 | (0.75-3.07) | 0.24 |
| Myometrial invasion |  |  |  |  |  |  |  |  |  |  |  |  |
| <1/2 vs. ≥1/2 | 0.27 | (0.16-0.47) | <0.01 | 0.68 | (0.33-1.38) | 0.28 | 0.28 | (0.14-0.54) | <0.01 | 0.46 | (0.20-1.03) | 0.06 |
| Cervical involvement |  |  |  |  |  |  |  |  |  |  |  |  |
| Positive vs. Negative | 2.85 | (1.56-5.20) | <0.01 | 1.25 | (0.64-2.43) | 0.51 | 3.14 | (1.56-6.30) | <0.01 | 1.86 | (0.87-3.98) | 0.11 |
| Ovarian metastasis |  |  |  |  |  |  |  |  |  |  |  |  |
| Positive vs. Negative | 4.93 | (2.64-9.19) | <0.01 | 1.19 | (0.54-2.63) | 0.67 | 7.30 | (3.69-14.46) | <0.01 | 1.13 | (0.44-2.91) | 0.79 |
| Lymph node metastasis |  |  |  |  |  |  |  |  |  |  |  |  |
| Positive vs. Negative | 4.91 | (2.87-8.42) | <0.01 | 3.27 | (1.69-6.32) | <0.01 | 1.97 | (0.94-4.14) | 0.07 |  |  |  |
| Distant metastasis |  |  |  |  |  |  |  |  |  |  |  |  |
| Positive vs. Negative | 14.00 | (7.63-25.71) | <0.01 | 13.25 | (5.73-30.65) | <0.01 | 16.00 | (8.27-30.95) | <0.01 | 10.30 | (4.10-25.83) | <0.01 |
| Lymphovascular invasion |  |  |  |  |  |  |  |  |  |  |  |  |
| Positive vs. Negative | 3.63 | (2.06-6.40) | <0.01 | 2.55 | (1.24-5.21) | 0.01 | 2.79 | (1.44-5.41) | <0.01 | 1.47 | (0.64-3.42) | 0.37 |
| Ascites or lavage cytology |  |  |  |  |  |  |  |  |  |  |  |  |
| Positive vs. Negative | 3.95 | (2.30-6.80) | <0.01 | 2.02 | (1.06-3.85) | 0.03 | 4.51 | (2.40-8.51) | <0.01 | 1.97 | (0.94-4.13) | 0.07 |
| Adjuvant therapy |  |  |  |  |  |  |  |  |  |  |  |  |
| Yes vs. No | 2.14 | (1.17-3.90) | 0.01 | 0.43 | (0.21-0.92) | 0.03 | 1.23 | (0.63-2.39) | 0.54 |  |  |  |
| Tumor-infiltrating lymphocytes |  |  |  |  |  |  |  |  |  |  |  |  |
| High vs. Low | 0.45 | (0.26-0.78) | <0.01 | 0.46 | (0.25-0.83) | <0.01 | 0.35 | (0.18-0.70) | <0.01 | 0.34 | (0.16-0.71) | <0.01 |

Abbreviations: HR, hazard ratio; CI, confidence interval.

**Table S10.** Univariate and multivariate analysis of progression-free survival and overall survival for cases with mismatch repair proficiency.

|  | Progression-free survival | | | | | | Overall survival | | | | | |
| --- | --- | --- | --- | --- | --- | --- | --- | --- | --- | --- | --- | --- |
|  | Univariate analysis | | | Multivariate analysis | | | Univariate analysis | | | Multivariate analysis | | |
| Variables | HR (95% CI) | | *p*-value | HR (95% CI) | | *p*-value | HR (95% CI) | | *p*-value | HR (95% CI) | | *p*-value |
| Age (years) |  |  |  |  |  |  |  |  |  |  |  |  |
| ≥60 vs. <60 | 3.09 | (1.88-5.07) | <0.01 | 2.38 | (1.41-4.01) | <0.01 | 2.77 | (1.43-5.37) | <0.01 | 2.16 | (1.06-4.37) | 0.03 |
| Performance status |  |  |  |  |  |  |  |  |  |  |  |  |
| 0, 1 vs. ≥2 | 0.14 | (0.04-0.43) | <0.01 | 0.19 | (0.06-0.67) | <0.01 | 0.18 | (0.02-1.33) | 0.09 |  |  |  |
| Histological subtypes |  |  |  |  |  |  |  |  |  |  |  |  |
| Aggressive type vs.  Non-aggressive type | 5.19 | (3.29-8.18) | <0.01 | 2.78 | (1.69-4.57) | <0.01 | 6.73 | (3.50-12.94) | <0.01 | 4.06 | (1.95-8.46) | <0.01 |
| Myometrial invasion |  |  |  |  |  |  |  |  |  |  |  |  |
| <1/2 vs. ≥1/2 | 0.23 | (0.15-0.36) | <0.01 | 0.63 | (0.35-1.13) | 0.12 | 0.24 | (0.13-0.45) | <0.01 | 0.59 | (0.25-1.37) | 0.22 |
| Cervical involvement |  |  |  |  |  |  |  |  |  |  |  |  |
| Positive vs. Negative | 1.94 | (1.14-3.28) | 0.01 | 1.26 | (0.70-2.27) | 0.44 | 1.26 | (0.56-2.84) | 0.58 |  |  |  |
| Ovarian metastasis |  |  |  |  |  |  |  |  |  |  |  |  |
| Positive vs. Negative | 6.55 | (3.92-10.97) | <0.01 | 2.71 | (1.40-5.27) | <0.01 | 10.31 | (5.36-19.83) | <0.01 | 3.46 | (1.42-8.42) | <0.01 |
| Lymph node metastasis |  |  |  |  |  |  |  |  |  |  |  |  |
| Positive vs. Negative | 5.50 | (3.45-8.75) | <0.01 | 2.24 | (1.26-4.01) | <0.01 | 6.09 | (3.26-11.36) | <0.01 | 2.27 | (1.05-4.92) | 0.04 |
| Distant metastasis |  |  |  |  |  |  |  |  |  |  |  |  |
| Positive vs. Negative | 11.89 | (6.67-21.18) | <0.01 | 5.23 | (2.57-10.62) | <0.01 | 10.89 | (5.12-23.18) | <0.01 | 2.97 | (1.13-7.79) | 0.03 |
| Lymphovascular invasion |  |  |  |  |  |  |  |  |  |  |  |  |
| Positive vs. Negative | 4.43 | (2.75-7.13) | <0.01 | 2.33 | (1.24-4.26) | <0.01 | 3.62 | (1.90-6.90) | <0.01 | 1.64 | (0.71-3.82) | 0.25 |
| Ascites or lavage cytology |  |  |  |  |  |  |  |  |  |  |  |  |
| Positive vs. Negative | 4.95 | (3.10-7.91) | <0.01 | 2.32 | (1.23-4.38) | <0.01 | 7.24 | (3.91-13.41) | <0.01 | 3.14 | (1.30-7.59) | 0.01 |
| Adjuvant therapy |  |  |  |  |  |  |  |  |  |  |  |  |
| Yes vs. No | 2.50 | (1.56-4.02) | 0.01 | 0.45 | (0.24-0.84) | 0.01 | 2.72 | (1.39-5.32) | <0.01 | 2.73 | (1.14-6.53) | 0.02 |
| Tumor-infiltrating lymphocytes |  |  |  |  |  |  |  |  |  |  |  |  |
| High vs. Low | 0.52 | (0.32-0.82) | <0.01 | 0.6 | (0.37-0.99) | 0.045 | 0.55 | (0.29-1.04) | 0.07 |  |  |  |

Abbreviations: HR, hazard ratio; CI, confidence interval.


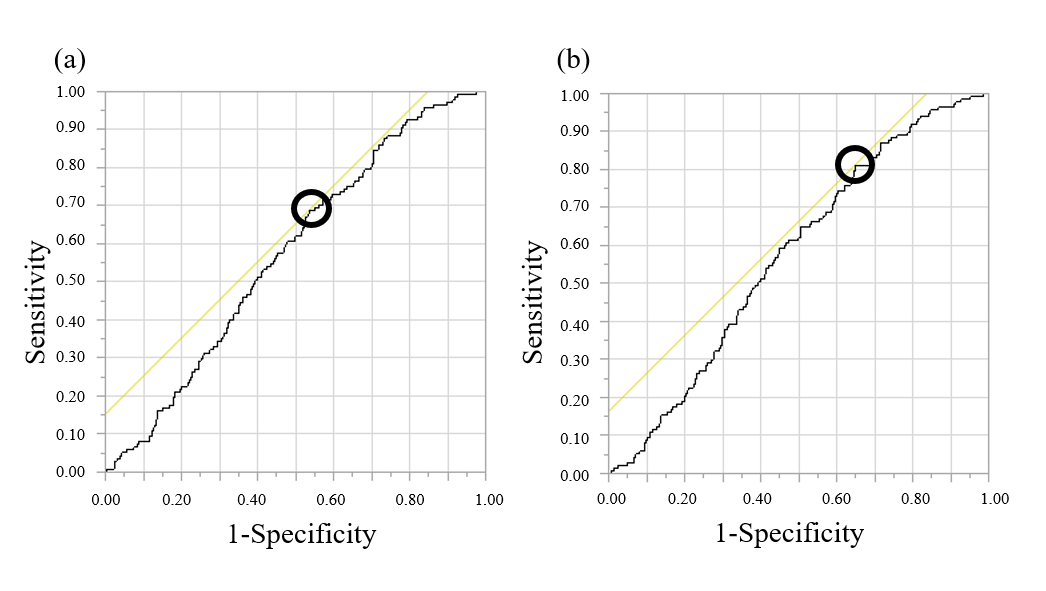


**Figure S1.** Receiver operating characteristics (ROC) curve analysis of the average number of lymphocytes per area (/μm^2^) based on artificial intelligence for recurrence or progression. (a) ROC curve analysis of the average number of lymphocytes per area (/μm^2^) within the range of 500 µm toward the stromal side from the invasive frontline for recurrence or progression. The area under the curve is 0.570 and the cutoff value was 0.00513. (b) ROC curve analysis of the average number of lymphocytes per area (/μm^2^) within the range of 250 µm toward the stromal side from the invasive frontline for recurrence or progression. The area under the curve is 0.573 and the cutoff value was 0.00639.


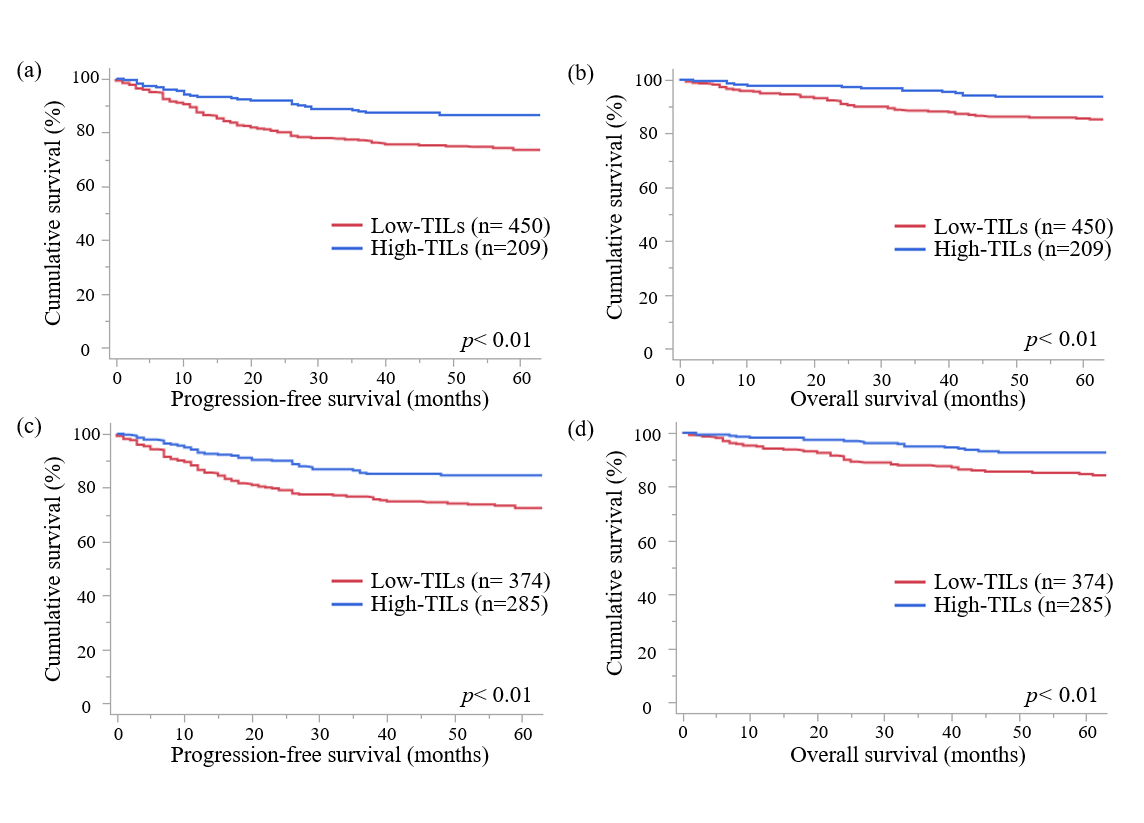


**Figure S2.** Survival analysis of the High-TIL and Low-TIL groups. (a) Progression-free survival (PFS) curves of all cases in the High-TIL and Low-TIL groups classified based on the area with the range of 500 µm toward the stromal side from the invasive frontline. The High-TIL group has a better prognosis than the Low-TIL group (*p*< 0.01). (b) Overall survival (OS) curves of all cases in the High-TIL and Low-TIL groups classified based on the area with the range of 500 µm toward the stromal side from the invasive frontline. The High-TIL group has a better prognosis than the Low-TIL group (*p*< 0.01). (c) PFS curves of all cases in the High-TIL and Low-TIL groups classified based on the area with the range of 250 µm toward the stromal side from the invasive frontline. The High-TIL group has a better prognosis than the Low-TIL group (*p*< 0.01). (d) OS curves of all cases in the High-TIL and Low-TIL groups classified based on the area with the range of 250 µm toward the stromal side from the invasive frontline. The High-TIL group has a better prognosis than the Low-TIL group (*p*< 0.01).


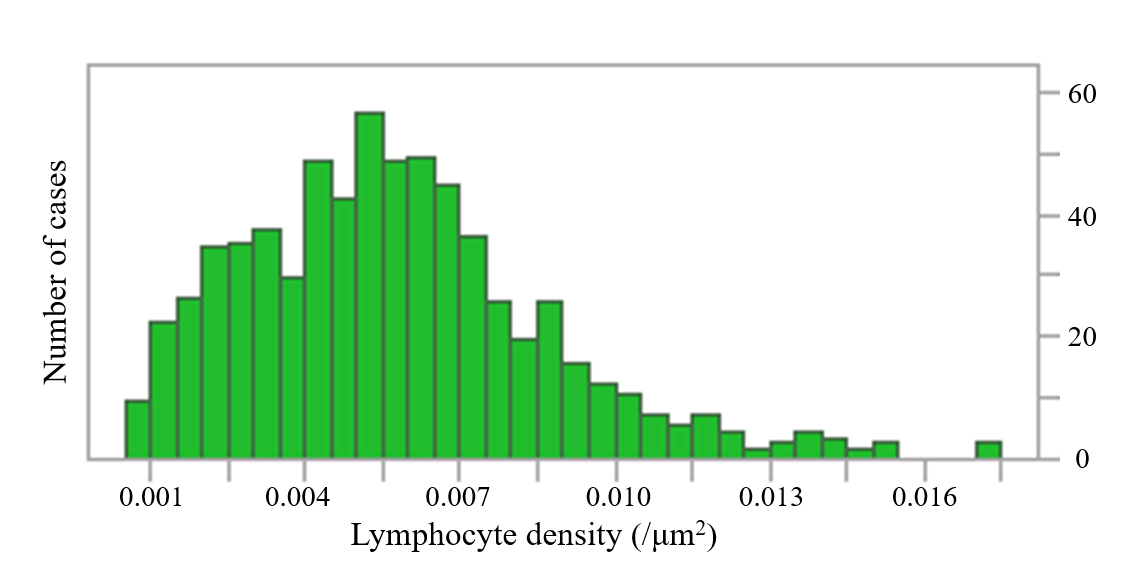


**Figure S3.** The lymphocytes density histogram. The first quartile, median, and third quartile numbers are 0.00301, 0.00493, and 0.00673, respectively


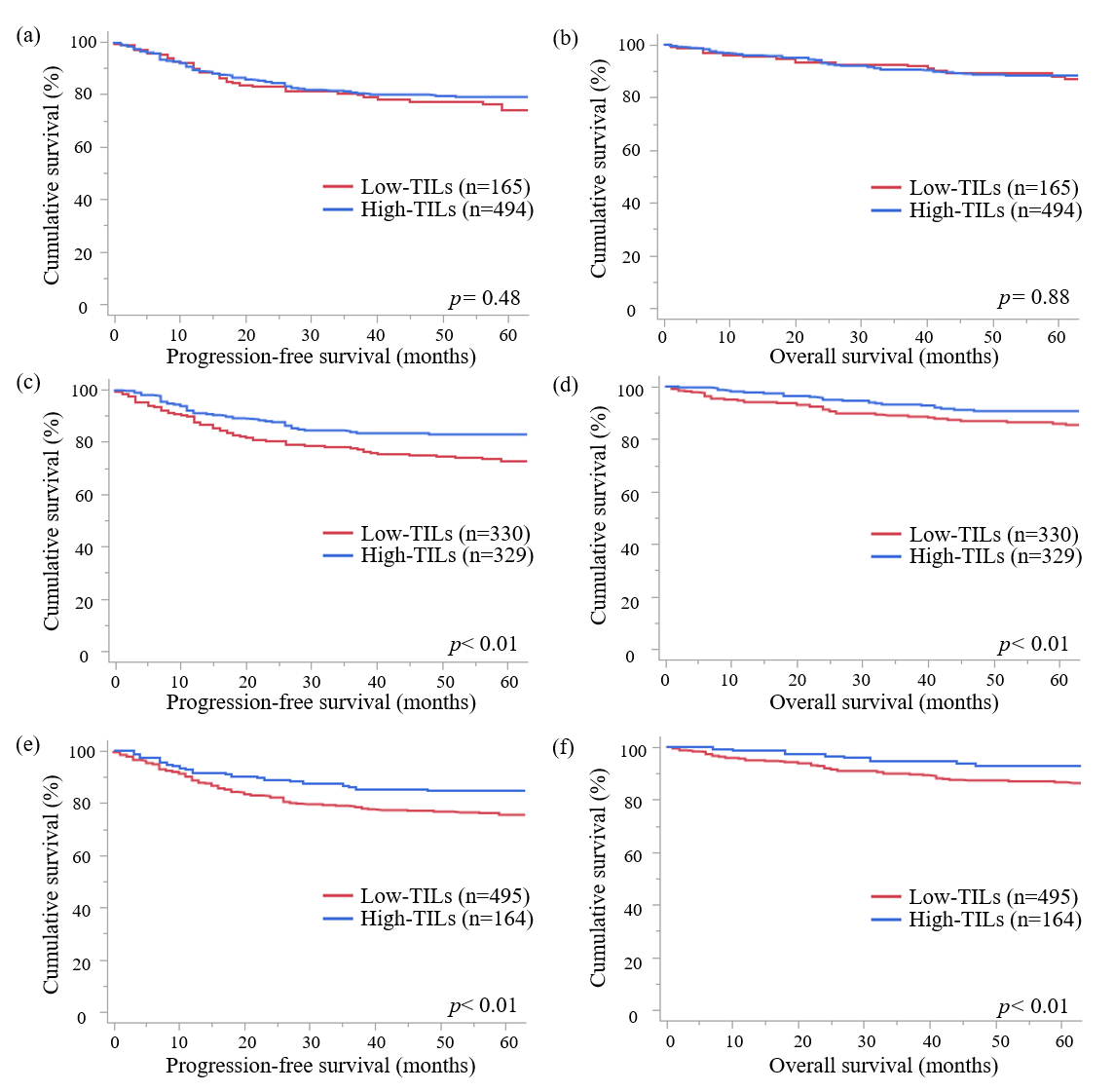


**Figure S4.** Survival analysis of the High-TIL and Low-TIL groups. (a) Progression-free survival (PFS) curves of all cases in the High-TIL and Low-TIL groups classified based on the first quartile number of the histogram of lymphocyte density. There is no prognostic significance between two groups (*p*= 0.48). (b) Overall survival (OS) curves of all cases in the High-TIL and Low-TIL groups classified based on the first quartile number of the histogram of lymphocyte density. There is no prognostic significance between two groups (*p*= 0.48). (c) PFS curves of all cases in the High-TIL and Low-TIL groups classified based on the median number of the histogram of lymphocyte density. The High-TIL group has a better prognosis than the Low-TIL group (*p*< 0.01). (d) OS curves of all cases in the High-TIL and Low-TIL groups classified based on the median number of the histogram of lymphocyte density. The High-TIL group has a better prognosis than the Low-TIL group (*p*< 0.01). (e) PFS curves of all cases in the High-TIL and Low-TIL groups classified based on the third quartile number of the histogram of lymphocyte density. The High-TIL group has a better prognosis than the Low-TIL group (*p*< 0.01). (f) OS curves of all cases in the High-TIL and Low-TIL groups classified based on the third quartile number of the histogram of lymphocyte density. The High-TIL group has a better prognosis than the Low-TIL group (*p*< 0.01).


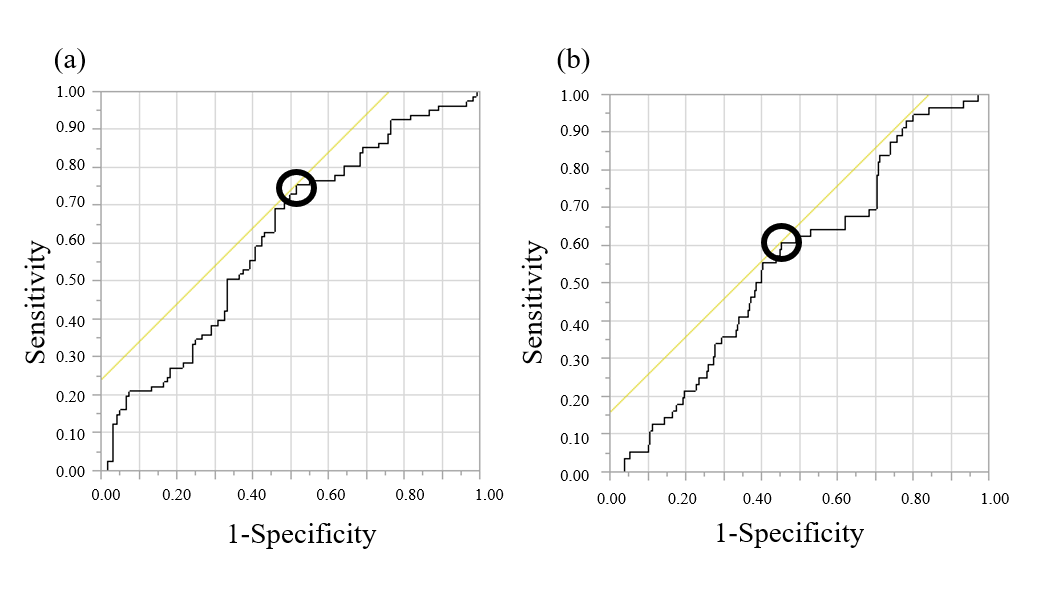


**Figure S5.** Receiver operating characteristics (ROC) curve analysis of the average number of lymphocytes per area (/μm^2^) based on artificial intelligence for recurrence or progression. (a) ROC curve analysis of the average number of lymphocytes per area (/μm^2^) for recurrence or progression in cases with aggressive type endometrial carcinoma. The area under the curve is 0.614 and the cutoff value was 0.00569. (b) ROC curve analysis of the average number of lymphocytes per area (/μm^2^) for recurrence or progression in cases with non-aggressive type endometrial carcinoma. The area under the curve is 0.555 and the cutoff value was 0.00478.


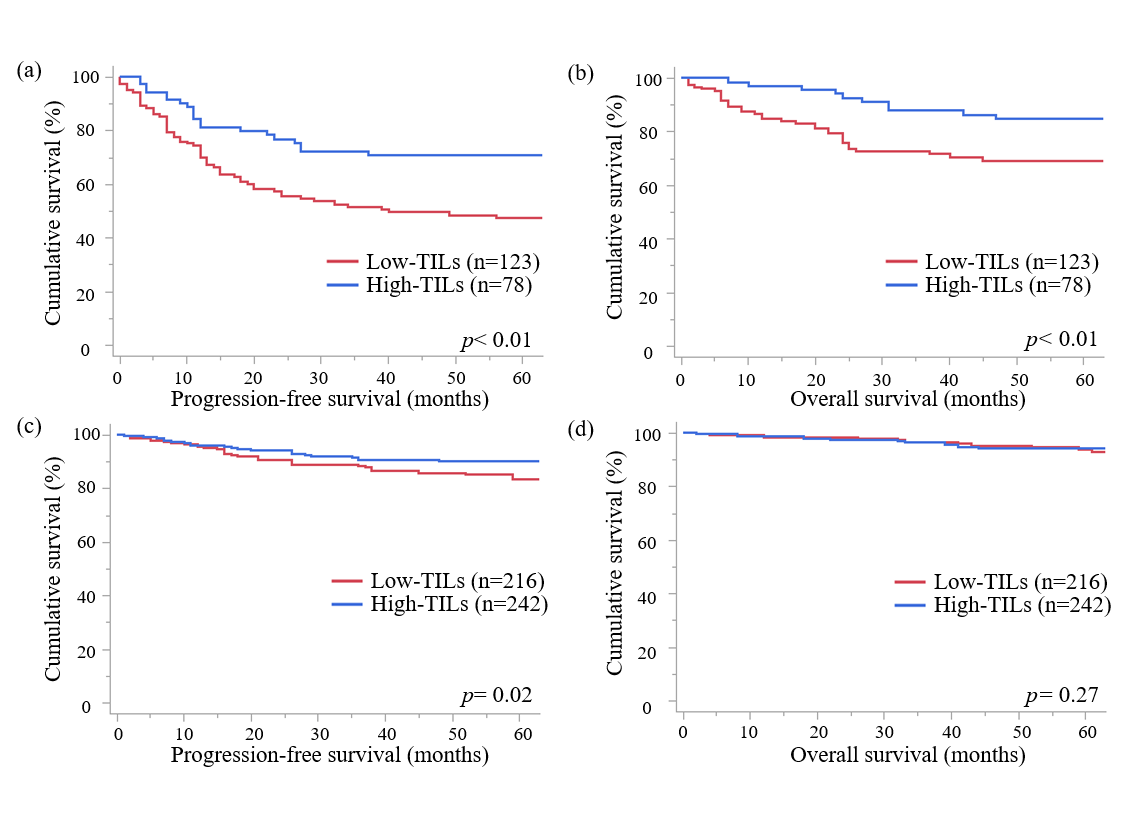


**Figure S6.** Survival analysis of the High-TIL and Low-TIL groups. (a) Progression-free survival (PFS) curves of all cases in the High-TIL and Low-TIL groups in cases with aggressive type endometrial carcinoma. The High-TIL group has a better prognosis than the Low-TIL group (*p*< 0.01). (b) Overall survival (OS) curves of all cases in the High-TIL and Low-TIL groups in cases with aggressive type endometrial carcinoma. The High-TIL group has a better prognosis than the Low-TIL group (*p*< 0.01). (c) PFS curves of all cases in the High-TIL and Low-TIL groups in cases with non-aggressive type endometrial carcinoma. The High-TIL group has a better prognosis than the Low-TIL group (*p*= 0.02). (d) OS curves of all cases in the High-TIL and Low-TIL groups in cases with non-aggressive type endometrial carcinoma. There is no prognostic significance between two groups (*p*= 0.27).


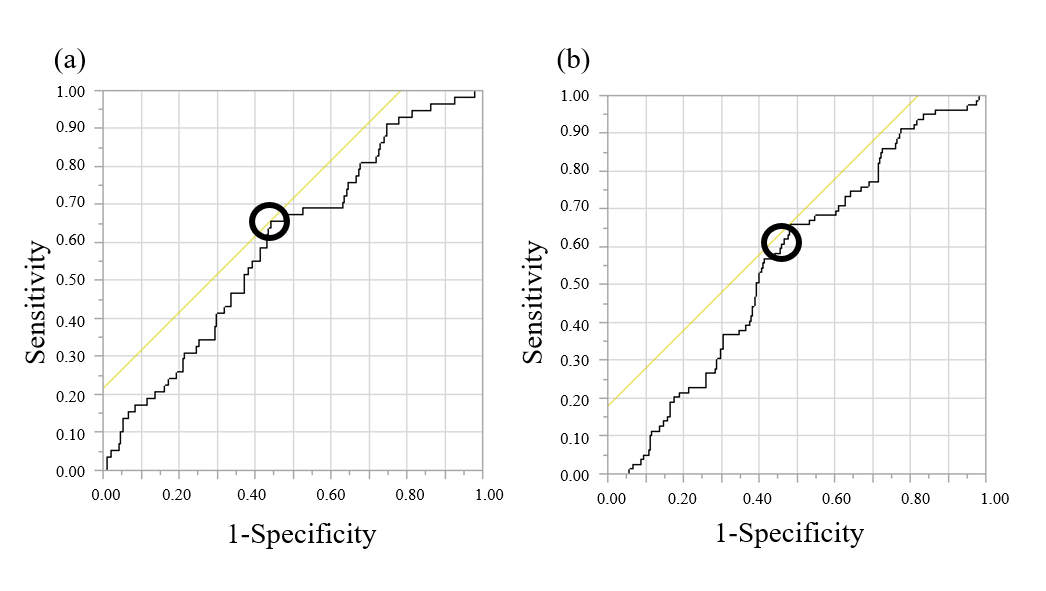


**Figure S7.** Receiver operating characteristics (ROC) curve analysis of the average number of lymphocytes per area (/μm^2^) based on artificial intelligence for recurrence or progression. (a) ROC curve analysis of the average number of lymphocytes per area (/μm^2^) for recurrence or progression in cases with mismatch repair deficiency. The area under the curve is 0.597 and the cutoff value was 0.00533. (b) ROC curve analysis of the average number of lymphocytes per area (/μm^2^) for recurrence or progression in cases with mismatch repair proficiency. The area under the curve is 0.558 and the cutoff value was 0.00478.


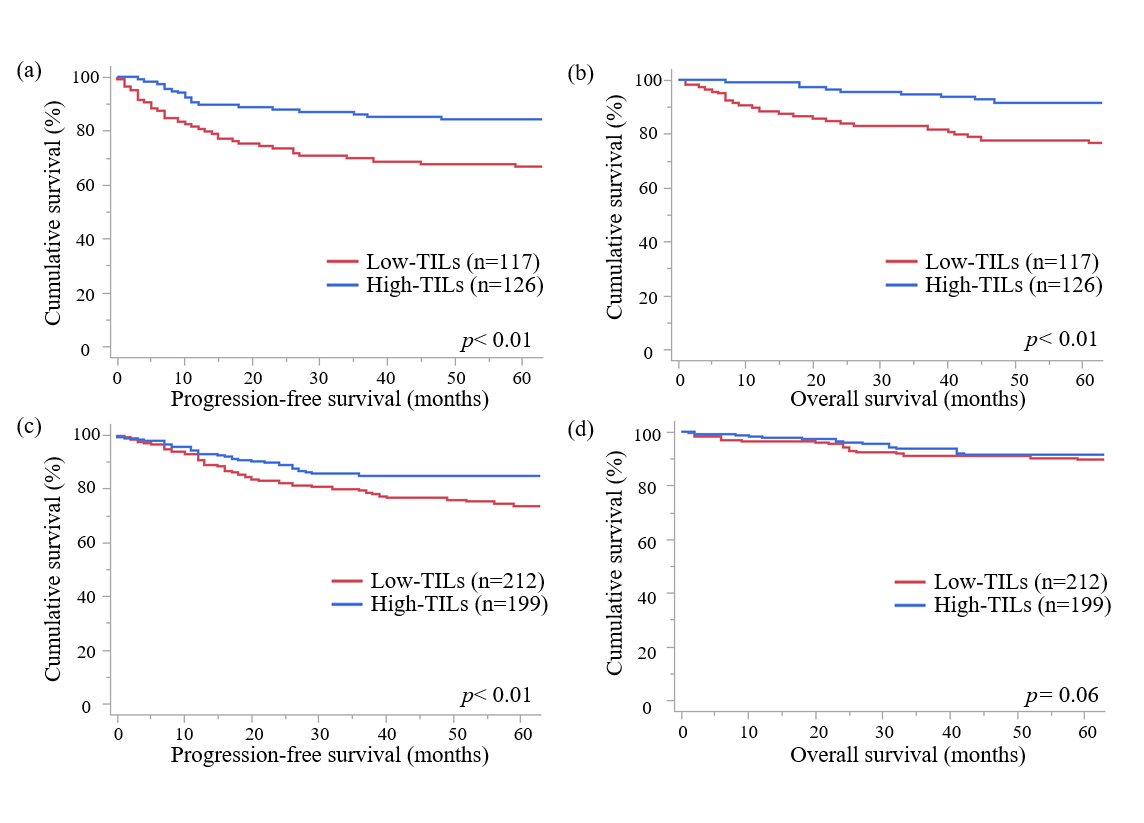


**Figure S8.** Survival analysis of the High-TIL and Low-TIL groups. (a) Progression-free survival (PFS) curves of all cases in the High-TIL and Low-TIL groups in cases with mismatch repair deficiency. The High-TIL group has a better prognosis than the Low-TIL group (*p*< 0.01). (b) Overall survival (OS) curves of all cases in the High-TIL and Low-TIL groups in cases with mismatch repair deficiency. The High-TIL group has a better prognosis than the Low-TIL group (*p*< 0.01). (c) PFS curves of all cases in the High-TIL and Low-TIL groups in cases with mismatch repair proficiency. The High-TIL group has a better prognosis than the Low-TIL group (*p*< 0.01). (d) OS curves of all cases in the High-TIL and Low-TIL groups in cases with mismatch repair proficiency. There is no prognostic significance between two groups (*p*= 0.06).
